# Causal relationships of infection with *Helicobacter pylori* and herpesvirus on periodontitis: A Mendelian randomization study

**DOI:** 10.1016/j.heliyon.2024.e35904

**Published:** 2024-08-06

**Authors:** Erli Wu, Ming Cheng, Shouxiang Yang, Wanting Yuan, Mengyun Gu, Dandan Lu, Lei Zhang, Qingqing Wang, Xiaoyu Sun, Wei Shao

**Affiliations:** a Stomatologic Hospital & College, Anhui Medical University, Key Lab. of Oral Diseases Research of Anhui Province, Hefei, 230032, China; b Arrail Dental Group, Beijing, 100012, China; c Department of Periodontology, Anhui Stomatology Hospital Affiliated to Anhui Medical University, Hefei, 230032, China; d Department of Microbiology and Parasitology, Anhui Provincial Laboratory of Pathogen Biology, School of Basic Medical Sciences, Anhui Medical University, Hefei, Anhui, China

**Keywords:** Periodontitis (PD), *Helicobacter pylori* (*H. pylori*) infection, Herpesvirus infection, Mendelian randomization, GWAS

## Abstract

**Background:**

To explore the causal association between *Helicobacter pylori* (*H. pylori*) infection, herpesvirus infection and periodontitis (PD) from a genetic perspective using Mendelian randomization (MR).

**Methods:**

The PD data were derived from genome-wide association study (GWAS) from the Dental Endpoints (GLIDE) consortium, and the FinnGen Biobank provided data on *H. pylori* and herpesvirus infections. In addition, we examined GWAS data for subtypes of *H. pylori* and herpesvirus infection. Inverse variance weighting (IVW) was selected as a major analysis technique, and weighted median (WM), weighted model, simple model, and MR-Egger regression were added as supplementary methods. To verify the findings, the effects of pleiotropy and heterogeneity were assessed.

**Results:**

Genetically predicted *H. pylori* infection (OR = 0.914, 95%CI = 0.693–1.205, P = 0.523), anti-*H. pylori* VacA (OR = 0.973, 95%CI = 0.895–1.057, P *=* 0.515), anti-*H. pylori* CagA (OR = 1.072, 95%CI = 0.986–1.164; P = 0.102), Epstein-Barr virus (EBV) infection (OR = 1.026, 95%CI = 0.940–1.120, P = 0.567), Herpes simplex virus (HSV) infection (OR = 0.962, 95%CI = 0.883–1.048, P = 0.372), cytomegalovirus (CMV) infection (OR = 1.025, 95%CI = 0.967–1.088, P = 0.415), EBV nuclear antigen-1 (EBNA1) (OR = 1.061, 95%CI = 0.930–1.209, P = 0.378), EBV virus capsid antigen (VCA) (OR = 1.043, 95CI% = 0.890–1.222, P = 0.603), HSV-1 (OR = 1.251, 95%CI = 0.782–2.001, P = 0.351), HSV-2 (OR = 1.020, 95%CI = 0.950–1.096, P = 0.585), CMV IgG (OR = 0.990, 95CI% = 0.882–1.111, P = 0.861) were not associated with PD, indicated that *H. pylori* and herpesvirus infection had no causal relationship to PD. Reverse studies also found no cause effect of PD on *H. pylori* or herpesvirus infection. The results of the sensitivity analysis suggested the robustness of the MR results.

**Conclusion:**

This study offered preliminary proof that *H. pylori* and herpesvirus infections were not causally linked to PD, and vice versa. However, more robust instrumental variables (IVs) and larger samples of GWAS data were necessary for further MR analysis.

## 1 Introduction

Periodontitis (PD) is a chronic oral disease characterized by plaque biofilm, causing deterioration of periodontal supporting tissues and the loss of teeth [1,2]. Due to its high incidence, PD has become the main cause of tooth loss in adults, which seriously affects the quality of life of patients [3]. According to epidemiological studies, mild to moderate PD affects 50 % of adults and has become the sixth greatest epidemic in human history [4].

The pathogenesis of PD is very complex, and the interaction between bacterial biofilm and the host immune system is the main factor in PD [5]. Some specific bacterial species, mainly gram-negative bacteria, such as *Porphyromonas gingivalis* (*P. gingivalis)*, *Fusobacterium nucleatum*, *Tannerella forsythia*, and *Treponema denticola*, are closely associated with PD [6]. In addition, with the development of molecular biology, microbiology and other disciplines, studies have shown that certain viruses may affect the development of PD. These viruses may reduce the host's resistance and lead to local immune dysfunction, which is conducive to the colonization of periodontal pathogens. Furthermore, periodontal infection caused by bacteria allows viruses to infect cells and enter the gum tissue, exacerbating the damage [7].

*Helicobacter pylori* (*H. pylori*) and herpesvirus are among the most prevalent bacteria and viruses in humans, respectively, and have a high infection rate in the population. All of them can be latent in the mouth and may participate in the progression of PD by interacting with other oral microorganisms and reducing host resistance [8,9]. Specifically, *H. pylori* is a type of gram-negative bacterium that specifically resides in the epithelium of the human stomach and affects around 60 % of people all over the world [10]. *H. pylori* is intimately connected to gastritis and gastric ulcers (GC), and long-term infection with *H. pylori* will raise the risk of gastric malignant tumors [11]. Additionally, *H. pylori* is also implicated as a potential risk factor for numerous oral conditions, like PD [12]. *P. gingivalis*, the major pathogen of PD, was found to be altered by pre-incubation with *H. pylori*, which altered the virulence of *P. gingivalis*, including hemagglutination, biofilm development, and bacterial internalization into oral keratinocytes [13]. Additionally, there was a substantial rise in IL-6, IL-8, and INF-γ following stimulation of a human leukemia mononuclear cell line with cagA + *H. pylori*, indicating that *H. pylori* can exacerbate the course of inflammation [14]. An increasing number of studies have documented the detection of *H. pylori* in the supringival and subgingival plaques of PD patients, offering compelling evidence for the correlation between *H. pylori* and PD [[15], [16], [17]]. Some surveys have also found that infection with *H. pylori* raises the incidence of PD, and eradicating *H. pylori* may alleviate the progression of PD [9,11].

Similarly, herpesvirus is the most common virus in humans, and research has indicated that herpesvirus is a significant contributor to the etiology of PD and that co-infection of the virus with periodontal bacteria may be a major cause of PD [8]. Herpes simplex virus (HSV), cytomegalovirus (CMV) and Epstein-Barr virus (EBV) are herpesviruses closely related to PD, which can aggravate and damage periodontal tissues by infecting immune cells of periodontal patients and promoting the increase of periodontal pathogens [18]. Multiple studies have demonstrated that the detection rates of HSV, CMV, and EBV are increased in patients with PD compared to healthy controls [[19], [20], [21]]. It was also observed that in cases of PD, the presence of herpesvirus increased with the seriousness of the disease [22,23]. More recently, the use of stage I therapy in cases of chronic PD can also lead to short-term elimination of the virus at the lesion site [24].

However, these results are based on observational studies. Observational studies have several limitations including detection bias, residual and unmeasured confusion, and reverse causality [25]. In addition, there are several reports that do not observe an association between these diseases [19,26]. More significantly, finding any correlation between *H. pylori* and herpesvirus infections and the risk of PD has significant clinical and public health implications due to the high prevalence of *H. pylori*, herpesvirus infections, and PD in the population, as well as the relative ease and cost-effectiveness of treatment against *H. pylori* and herpesvirus infections. For these reasons, better methods are needed to assess the causal effects of *H. pylori* and herpesvirus infection on the pathogenesis of PD. Therefore, improved methods are required to evaluate the causal relationships between these diseases.

Mendelian randomization (MR), an epidemiology technique, uses multiple single-nucleotide polymorphisms (SNPs) as genetic instrumental variables (IVs) to evaluate causality in a proposed exposure-outcome pathway. Since SNPs are distributed at random during conception, it can prevent reverse causality and other confounding consequences [27]. In this study, we assessed the causality of *H. pylori* and herpesvirus infection for PD by using MR analysis. Also, a reverse MR study was also performed.

## 2 Materials and methods

### 2.1 Study design and materials

**Fig. 1:**
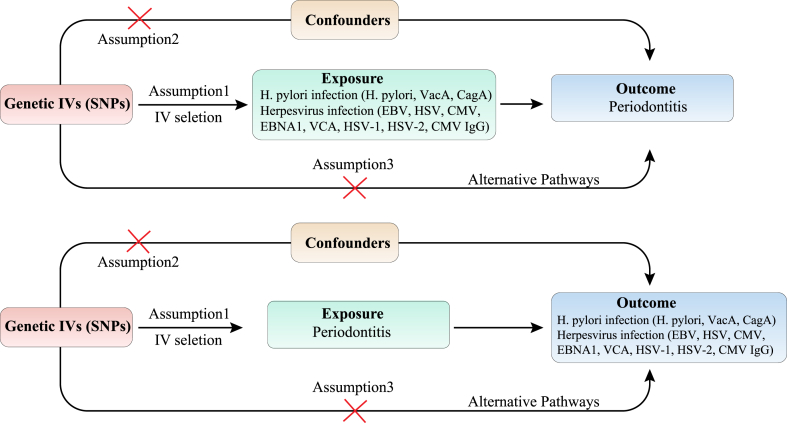
Flowchart of this MR analysis. Abbreviations: SNPs, single-nucleotide polymorphisms; *H. pylori*, *Helicobacter pylori*; VacA, Vacuolar cytotoxin A; CagA, Cytotoxin-associated protein A; EBV, Epstein-Barr virus; HSV, Herpes simplex virus; CMV, cytomegalovirus; EBNA1, EBV nuclear antigen-1; VCA, EBV virus capsid antigen; HSV-1, HSV type 1; HSV-2, HSV type 2.

SNPs indicating genetic variation were chosen as instrumental variables (IVs) for two-sample MR analysis. IVs were required to fulfill three conditions: (1) IVs have a strong and direct relationship with exposure; (2) IVs are not subject to confounding factors; (3) IVs exclusively influence outcomes through exposure [28,29]. The causality of *H. pylori* and herpesvirus infection on PD was evaluated through MR analysis. Subsequently, we performed a reverse MR analysis with PD as the exposure and *H. pylori* and herpesvirus infection as the outcomes. Fig. 1 showed a schematic of the MR study between *H. pylori* infection, herpesvirus infection, and PD.

### 2.2 Data source

The latest meta-analysis of GWAS from the Gene-Lifestyle Interaction in the Dental Endpoints (GLIDE) consortium provided summary statistics for PD. This study has the biggest sample size to date, consisting of 28,210 controls and 17,353 clinically diagnosed cases [30]. The Community Periodontal Index (CPI) or the Centers for Disease Control and Prevention/American Academy of Periodontology (CDC/AAP) case definitions were used to categorize PD cases. Alternatively, the number of deep periodontal pockets and the depth of probing were utilized to determine the PD diagnostic report of study participants [30]. The European Bioinformatics Institute database, which comprised 1058 patients and 3625 healthy individuals, provided the GWAS summary information for *H. pylori* infection. Samples from both databases were of European descent. In addition, we also collected GWAS data on subtypes of infection with *H. pylori* to explore the causation between infection with *H. pylori* and PD, which included anti-*H. pylori* VacA and anti-CagA. Aggregated GWAS statistics for anti-*H. pylori* VacA and anti-CagA were from the UK Biological Sample Database, which included 1571 and 985 samples, respectively. Moreover, we used the GWAS data from the FinnGen biobank analysis for herpesvirus infection. In the latest version, EBV infection (R10) included 2979 cases and 400,974 controls; HSV infection (R10) included 3723 cases and 396,378 controls; CMV (R7) included 428 cases and 301,439 controls. Besides these, we also used GWAS data on circulating antibody IgG levels associated with several herpesvirus subtypes including EBV nuclear antigen-1 (EBNA1), EBV virus capsid antigen (VCA), CMV, HSV type 1 (HSV-1) and HSV type 2 (HSV-2). The samples were collected from the Milieu Interieur cohort, which comprises 1000 healthy individuals who were tested with a 2200 IgG kit for sero-specific IgGTM against various herpevirus antigens [31]. Table 1 displayed the specifics of the GWAS data used in this study.

**Table 1:** Details of the studies in the mendelian randomization analysis.

Phenotype	Consortium	Population	Sample size	Cases	Controls	Access Link
PD	GLIDE	European	45,563	17,353	28,210	https://data.bris.ac.uk/data/dataset/2j2rqgzedxlq02oqbb4vmycnc2
Anti-*H. Pylori* IgG levels	EBI	European	4683	1058	3625	https://gwas.mrcieu.ac.uk/datasets/ieu-b-4905/
*H. pylori* VacA antibody levels		European	1571	–	–	https://gwas.mrcieu.ac.uk/datasets/ebi-a-GCST90006916/
*H. pylori* CagA antibody levels		European	985	–	–	https://gwas.mrcieu.ac.uk/datasets/ebi-a-GCST90006911/
EBV infection	FinnGen cohort	European	403,953	2979	400,974	https://r10.finngen.fi/
HSV infection		European	400,101	3723	396,378	https://r10.finngen.fi/
CMV infection		European	301,867	428	301,439	https://r7.finngen.fi/
EBNA1 IgG	Milieu Intérieur cohort	European	1000	914	86	https://doi.org/10.5281/zenodo.1217136
VCA IgG		European	1000	956	44	
HSV-1 IgG		European	1000	645	355	
HSV-2 IgG		European	1000	208	792	
CMV IgG		European	1000	347	653	

Abbreviations: *H. pylori, Helicobacter pylori*; VacA, Vacuolar cytotoxin A; CagA, Cytotoxin-associated protein A; PD, periodontitis; GLIDE, Gene-Lifestyle Interaction in the Dental Endpoints; EBI, European Bioinformatics Institute; EBV, Epstein-Barr virus; HSV, herpes simplex; CMV, cytomegalovirus; EBNA1, Epstein-Barr virus nuclear antigen-1; VCA, EBV viral capsid antigen; HSV-1, HSV type 1; HSV-2, HSV type 2.

### 2.3 IV selection

Genetic IV can be acquired through GWAS aggregated data analysis or a literature search. The *H. pylori* infection's genetic IV was provided by a prior investigation carried out by Mayerle et al. [32]. Two SNPs, rs10004195 and rs368433, were shown to be independently and highly linked with seropositivity for *H. pylori* in their investigation of a European population, which comprised 2763 cases and 8175 controls, as indicated in Table S1. Consequently, these 2 SNPs were chosen for IVs linked to *H. pylori* infection and were employed in subsequent analyses. For other GWAS summary data, to satisfy three assumptions of MR analysis, IVs were obtained in accordance with *p* < 5 × 10^−6^ to obtain more SNPs. Then, we removed SNPs that have a substantial linkage imbalance (LD) by filtering them out with the aggregation software *R*^*2*^ *=* 0.001*,* clumping distance = 10,000 kb [33]. Additionally, each SNP's *F* statistic is determined using the following equation: (1) *F* = *R*^2^ × (N-2)/(1-*R*^2^) or (2) *F* = beta^2^/se^2^. *R*^2^ is a representation of the exposure variability for each IV interpretation [34,35]. An IV will not be included in the MR analysis if its *F* statistic is less than 10, which is regarded as a poor instrument. In order to confirm the second MR hypothesis, we scanned the PhenoScannerV2 database for IV and its proxy qualities and excluded SNPs that may affect the outcome due to other confounding variables [36,37]. Finally, palindrome SNPs were omitted to avoid the effect of alleles on the outcome of the causal association between the diseases [38].

### 2.4 MR analysis

The study was statistically analyzed using R (version 4.3.1). For MR analysis, the "TwoSampleMR” package in R was used to find any potential causative relationships between *H. pylori* infection, herpesvirus infection and PD [39]. Inverse variance weighting (IVW) was employed in the main study [40]. As additional techniques for MR analysis, weighted median, weighted models, simple models, and MR-Egger regression were applied [41]. The 95 % confidence interval (CI) and odds ratio (OR) serve as the main effect measures in this investigation. If OR>1, it is demonstrated that the factor can encourage the outcome to occur; if not, the reverse [42]. P < 0.05 was used as the significance criterion to detect plausible causal effects.

### 2.5 Sensitivity analysis

After MR analysis, the Cochran's Q test was employed to assess heterogeneity in the IVW and MR Egger regression methods. If P > 0.05, we considered that the results of the study were not affected by heterogeneity [24]. The Pleiotropy Residual Sum and Outlier (MR PRESSO) and MR-Egger methods were used to analyze the pleiotropy between SNPs as instrumental factors [43]. If P > 0.05, pleiotropy was not present in causal analysis, and its effects may be ignored. The MR-Egger regression can identify and correct pleiotropy [44]. Furthermore, the MR-PRESSO approach could identify whether horizontal pleiotropy exists based on a global test. If it is found, it offers estimates derived from this analysis that are adjusted for horizontal pleiotropy by eliminating potential outliers [45]. Additionally, a leave-one-out approach was used, systematically excluding each SNP throughout the MR analysis to identify any SNPs that potentially had an impact [46].

## 3 Results

### 3.1 Causal effect of *H. pylori* infection on PD

**Fig. 2:**
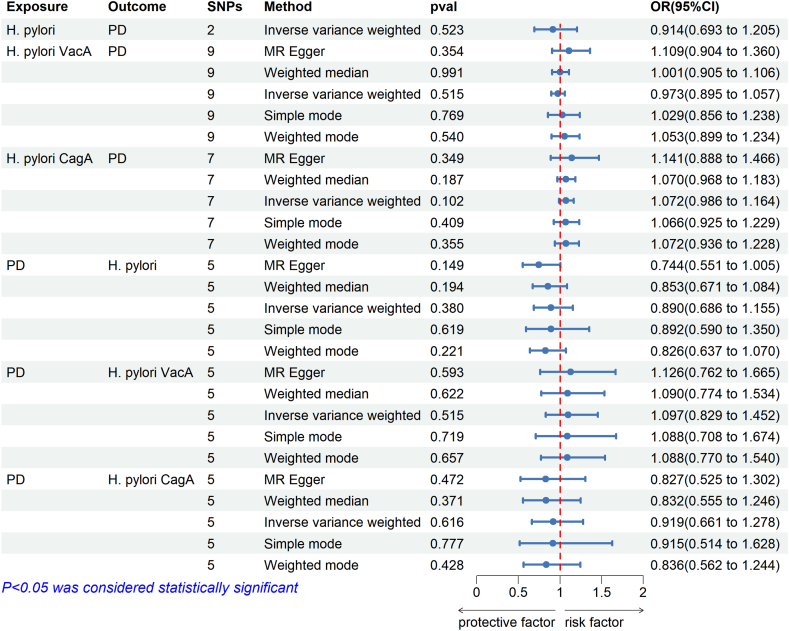
Causal estimates between *H. pylori* infection and PD given as odds ratios (ORs) and 95 % confidence intervals. SNPs, single-nucleotide polymorphisms; *H. pylori*, *Helicobacter pylori*; OR, Odds ratio; 95 % CI, 95 % confidence intervals; VacA, Vacuolar cytotoxin A; CagA, Cytotoxin-associated protein A; PD, periodontitis.

The MR analysis results were displayed in Fig. 2 rs10004195 and rs368433, two SNPs highly connected to *H. pylori* infection, had *F*-statistics of 558.639 and 289.986, respectively, which prevented weak IVs from affecting causality. MR analysis revealed that IVW (OR = 0.914, 95%CI = 0.693–1.205, P = 0.523), indicating that *H. pylori* infection had no effect on PD.

In addition, analysis was performed on the two main subtypes of *H. pylori* infection: *H. pylori* VacA antibody and *H. pylori* CagA antibody. 9 and 7 SNPs were eventually accepted to analyze the causal effect of VacA and CagA on PD in the MR study. In Tables S2–S3, the IVs used for MR analyses were fully described. The MR estimates showed no causal impact of VacA (OR = 0.973, 95%CI = 0.895–1.057, P = 0.515) and CagA (OR = 1.072, 95%CI = 0.986–1.164, P = 0.102) on PD. The findings from the WM and MR Egger tests were in agreement with IVW methods.

The results of the sensitivity analysis showed no heterogeneity or directional pleiotropy in VacA and CagA studies on PD, indicating that our MR results were robust (Table 2). The leave-one-out analysis showed no outliers existed, suggesting that a specific SNP did not impact the findings. The scatter plots, leave-one-out analyses, and funnel plots of the MR analyses were displayed in Figs. S1–S3.

**Table 2:** The sensitivity analysis of MR study between *H. pylori* infection and PD.

Exposure	Outcome	SNPs	IVW		MR-Egger regression		MRPRESSO	
			Q statistic	Q_pval	Interce pt	P-value	RSSobs	P-value
*H. pylori*	PD	2	8.519	0.004	–	–	–	–
VacA	PD	9	11.516	0.174	−0.037	0.215	20.786	0.175
CagA	PD	7	7.051	0.316	−0.021	0.622	12.360	0.348
PD	*H. Pylori* IgG	5	8.173	0.085	0.044	0.192	21.372	0.054
	VacA	5	0.173	0.996	−0.006	0.865	0.468	1.000
	CagA	5	1.223	0.874	0.028	0.554	13.199	0.256

Abbreviations: MR, Mendelian randomization; SNPs, single-nucleotide polymorphisms; IVW, inverse variance-weighted method; *H. pylori*, *Helicobacter pylori*; VacA, Vacuolar cytotoxin A; CagA, Cytotoxin-associated protein A; PD, periodontitis.

### 3.2 Causal effect of PD on *H. pylori* infection

8 SNPs as IVs were identified for PD after filtering out weak IVs and LD. They are all highly correlated with exposure (*F* > 10). Then, by combining the outcome database and removing SNPs that were palindromic, 5 SNPs were approved for MR estimates of PD on IgG for *H. pylori* infection, anti-*H. pylori* VacA, and anti-CagA. Table S4 showed the details of the SNPs associated with PD.

We did not find an impact of PD on anti*-H. pylori* IgG levels (OR = 0.890, 95%CI = 0.686–1.155; P = 0.380), anti-*H. pylori* VacA (OR = 1.097, 95%CI 0.829–1.452; P = 0.515) and anti-*H. pylori* CagA (OR = 0.919, 95%CI = 0.661–1.278, P = 0.615). The findings from the WM and MR Egger tests were in agreement with IVW.

The sensitivity analysis confirmed the absence of horizontal pleiotropy or heterogeneity. No abnormal SNPs were observed in both the leave-one-out MR analysis and the MR-PRESSO regression methods.

### 3.3 Causal effect of herpesvirus infection on PD

58 SNPs were used for herpesvirus infection on PD after filtering, which all have an F statistic greater than 10 (ranging from 20.983 to 47.626). Table S5 showed the details of the SNPs associated with the herpesvirus.

**Fig. 3:**
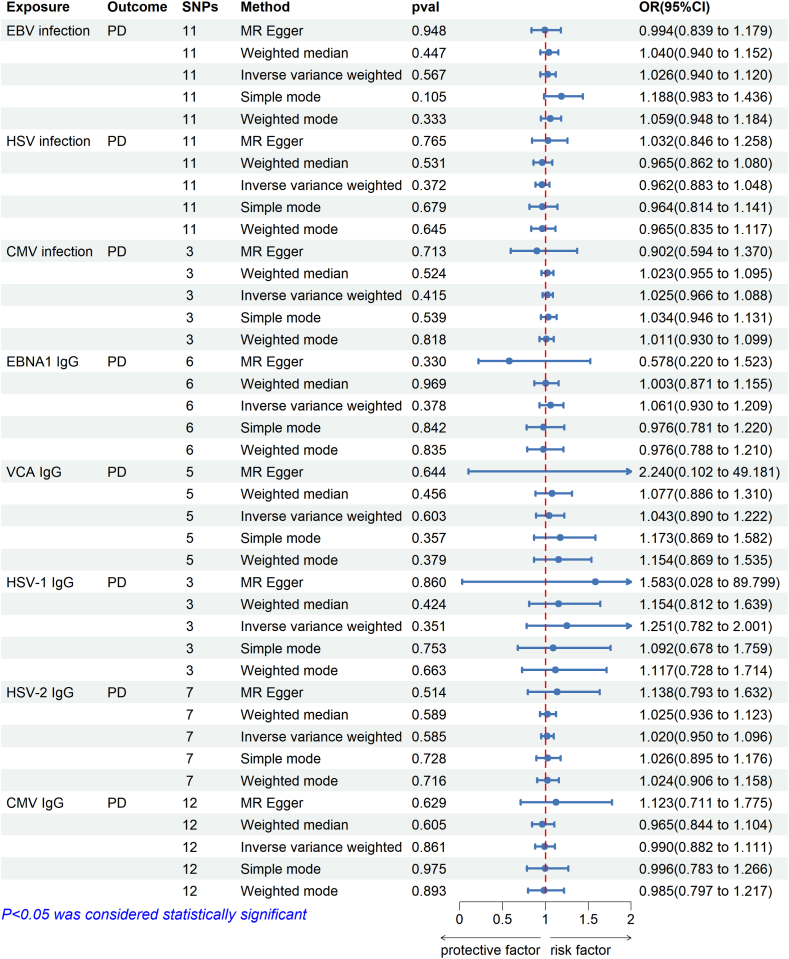
Causal estimates between herpesvirus infection and PD given as odds ratios (ORs) and 95 % confidence intervals. SNPs, single-nucleotide polymorphisms; *H. pylori*, *Helicobacter pylori*; OR, Odds ratio; 95 % CI, 95 % confidence intervals; EBV, Epstein-Barr virus; HSV, Herpes simplex virus; CMV, cytomegalovirus; EBNA1, EBV nuclear antigen-1; VCA, EBV virus capsid antigen; HSV-1, HSV type 1; HSV-2, HSV type 2; PD, periodontitis.

Fig. 3 illustrated the effect between each herpesvirus and PD using the IVW approach. Specifically, in the FinnGen cohort, genetically predicted EBV infection (OR = 1.026, 95%CI = 0.940–1.120, P = 0.567), HSV infection (OR = 0.962, 95%CI = 0.883–1.048, P = 0.372), and CMV infection (OR = 1.025, 95%CI = 0.967–1.088, P = 0.415) were not associated with PD risk. In the Milieu Interieur cohort, we found no correlations between herpesvirus-related IgG EBNA1 (OR = 1.061, 95%CI = 0.930–1.209, P = 0.378), VCA (OR = 1.043, 95CI% = 0.890–1.222, P = 0.603), HSV-1 (OR = 1.251, 95%CI = 0.782–2.001, P = 0.351), HSV-2 (OR = 1.020, 95%CI = 0.950–1.096, P = 0.585), CMV (OR = 0.990, 95CI% = 0.882–1.111, P = 0.861) and PD risk.

In the sensitivity analysis, according to the Cochran's Q test, no heterogeneity was detected between the instrumental SNP effects of the herpesvirus, except for HSV-1. Therefore, the random effects model was used for the MR analysis of HSV-1. Additionally, the MR-Egger intercept and MR-PRESSO global tests revealed no indication of horizontal pleiotropy (Table 3). The leave-one analysis, forest plot, and scatter plot showed that our results are reliable and consistent with our earlier testing (Figs. S4–S6).

**Table 3:** The sensitivity analysis of MR study between herpesvirus infection and PD.

Exposure	Outcome	SNPs	IVW		MR-Egger regression		MRPRESSO	
			Q statistic	Q_pval	Interce pt	P-value	RSSobs	P-value
EBV infection	PD	11	16.720	0.081	0.008	0.678	23.025	0.127
HSV infection		11	5.625	0.846	−0.013	0.462	6.510	0.914
CMV infection		3	0.713	0.700	0.054	0.653	1.877	0.797
EBNA1 IgG		6	7.892	0.162	0.101	0.283	11.467	0.267
VCA IgG		5	5.266	0.261	−0.083	0.660	7.798	0.395
HSV-1 IgG		3	6.671	0.036	−0.024	0.927	10.473	0.154
HSV-2 IgG		7	2.691	0.846	−0.022	0.571	3.864	0.873
CMV IgG		12	17.671	0.090	−0.025	0.587	20.671	0.270
PD	EBV infection	6	11.207	0.047	−0.020	0.569	15.431	0.263
	HSV infection	6	2.196	0.821	−0.008	0.693	4.335	0.877
	CMV infection	6	1.948	0.856	0.004	0.945	8.001	0.674
	EBNA1 IgG	3	0.626	0.731	0.007	0.920	1.848	0.878
	VCA IgG	3	3.268	0.195	−0.013	0.900	15.699	0.071
	HSV-1 IgG	3	1.961	0.375	−0.020	0.714	13.010	0.125
	HSV-2 IgG	3	0.543	0.762	−0.030	0.741	3.440	0.711
	CMV IgG	2	17.671	0.090	−0.025	0.587	20.671	0.270

Abbreviations: MR, Mendelian randomization; SNPs, single-nucleotide polymorphisms; IVW, inverse variance-weighted method; EBV, Epstein-Barr virus; HSV, Herpes simplex virus; CMV, cytomegalovirus; EBNA1, EBV nuclear antigen-1; VCA, EBV virus capsid antigen; HSV-1, HSV type 1; HSV-2, HSV type 2; PD, periodontitis.

### 3.4 Causal effect of PD on herpesvirus infection and subtypes

In the reverse MR study, we also did not find an association between PD and herpesvirus infection. In particular, the *p*-value of PD for all herpesvirus infections and their subtypes was greater than 0.05, suggesting that there is no causal effect of PD on herpesvirus infections (Fig. S7). Table S6 documented the IVs associated with PD for herpesvirus infection.

Except for EBV infection, which had a P-value of less than 0.05 and thus used a random effects model, the heterogeneity test results were all P > 0.05, which meant no heterogeneity effect in the results of MR analysis. We performed MR PRESSO to check for horizontal pleiotropy, and the results were all P > 0.05, which indicated there was no effect of horizontal pleitropy. A leave-one-out test was also performed to test each SNP and the results indicated that it was unlikely that a single SNP affected the MR results. Therefore, the results of the MR analysis were believed to be reliable and stable.

## 4 Discussion

In this study, we explored the causal effect of *H. pylori* infection and herpesvirus infection on PD using MR methods. The findings of the MR study did not uncover any proof linking *H. pylori* and herpesvirus infections to PD. The results of the reverse MR study were consistent with the aforementioned results. The results of the sensitivity analysis suggested the reliability and robustness of our MR analysis results.

The oral cavity, as the entrance and initial component of the gastrointestinal system, has been generally recognized by researchers as a primary extra-gastric reservoir of *H. pylori* [47,48]. Therefore, *H. pylori* is also thought to be a possible bridge between gastrointestinal diseases and PD. A meta-analysis reported a strong association between the presence of *H. pylori* in the oral cavity and in the stomach [49]. Other studies have found that 83.3 % of the patients with *H. pylori* in the oral cavity were also positive for the bacterium in gastric samples [50]. In addition, poor oral hygiene and microaerobic conditions caused by chronic PD infections favor *H. pylori* colonization of the periodontal pouch, suggesting that even if *H. pylori* is eradicated from the stomach, dental plaque can become a sanctuary for *H. pylori* and have an impact on re-infection, leading to a recurrence of stomach ulcers [51,52]. A recent MR study also suggested that PD was associated with an increased risk of GC, and the pathway may be associated with *H. pylori* in oral [53]. Moreover, the occurrence of oral *H. pylori* infections is directly related to the oral hygiene and periodontal health status of an individual, and the improvement of oral hygiene and oral health may increase the inhibition rate of gastric *H. pylori* infection [54]. The above-mentioned studies suggest that there may be a reciprocal relationship between *H. pylori*-induced PD and *H. pylori*-induced GC.

Some observational studies have examined the links between infection with *H. pylori* and PD. According to research including 14 individuals with chronic PD, patients who had *H. pylori* had more loss of attachment and deeper probing depth than those who did not [14]. Another cross-sectional study involving 53 volunteers also discovered a relationship between the existence of PD and genotypes and rates of detection of *H. pylori* in subgingival plaque [55]. Riggio et al. [15] identified *H. pylori* in the sub-gingival plaque of adults with PD through the PCR technique. Additionally, a study involving 4955 participants between the ages of 20 and 90 who underwent periodontal exams and serum testing for *H. pylori* was conducted to examine a correlation between PD and infection with *H. pylori*, suggesting a strong connection between PD and a higher probability of infection with *H. pylori* in comparison to good periodontal health (OR = 1.271, 95 % CI = 1.177–1.372) [12]. Furthermore, a case-control study showed that adults with poorer oral hygiene exhibited greater *H. pylori* levels detected in stomachs and dental plaque [56]. Moreover, a meta-analysis involving 2727 participants from Asia revealed a connection between oral *H. pylori* infection and PD (OR = 2.53; 95%CI = 1.86–3.44, P < 0.05) [47]. Another meta-analysis including eight studies suggested that the possibility of *H. pylori* infection was 2.47 times higher in individuals with PD (OR = 2.47; 95%CI = 2.01–3.03; P = 0.001) [57]. In addition, two meta-analyses reported that combined periodontal treatment can reduce gastric *H. pylori* rates [58,59]. Nevertheless, certain research findings showed no association between *H. pylori* infection and PD. A case-control study involving 134 participants showed no correlation between infection with *H. pylori* and patients with poor oral hygiene and PD [26]. Another case-control study involving 100 participants revealed no significant correlation between *H. pylori* presence in gingival crevicular fluid and PD status (P = 0.200) [60]. Moreover, a meta-analysis including four observational studies indicated no links between *H. pylori* and PD [61]. Furthermore, a study involving 959 healthy Japanese adults found that *H. pylori* infection was positively correlated with tooth loss in males, but negatively correlated with the prevalence of PD in females [62].

Herpesvirus infection is another disease with a high prevalence in the population, which may influence the development of PD by reducing the resistance of periodontal tissues and decreasing host resistance to periodontal pathogens [63]. A study by Contreras et al. [64] using nested polymerase chain reaction in 15 human samples indicated an increased detection rate of CMV and EBV-1 in PD lesions than that in healthy periodontal sites. According to a case-control study involving 20 healthy participants, 40 gingivitis patients, and 40 patients with chronic PD, the levels of HSV-1 and EBV detection in chronic PD patients were considerably greater than those in the healthy controls (P < 0.001). Furthermore, a positive correlation was seen between the incidence of EBV in patients suffering from chronic PD and a rise in gingival index, probing depth, and loss of clinical attachment (P < 0.05) [65]. A meta-analysis showed a significant correlation between EBV, CMV and PD [66]. Moreover, Kubar et al. [67] discovered that invasive PD lesions had a greater incidence of CMV and EBV DNA copies in the gum tissue and periodontal pocket than did chronic PD lesions, indicating a possible correlation between the herpesvirus count and the severity of PD. Puletic et al. [68] also collected subgingival samples from 39 periodontal abscesses, 33 necrotizing ulcerative PD, 27 chronic PD and 30 healthy controls, conducted PCR analysis for microbial detection, and found higher detection rates of EBV and CMV in severe PD patients than those in healthy subjects and patients with moderate PD. Nevertheless, some studies have shown no relationship between the herpesvirus and the prevalence of PD. Nibali et al. [19] performed PCR analysis to detect CMV and EBV in 140 ethnically mixed (predominantly Caucasian) subjects including 16 affected by limited invasive PD, 64 affected by generalized aggressive PD, 20 affected by chronic PD, and 40 healthy controls. The sample size of this study was much larger than the other studies, and the results of this study found that CMV DNA was undetectable in all plaque samples and only a small percentage of PD patients and healthy controls carried EBV. Dawson et al. [69] found a rare rate of both CMV and EBV in the subgingival region of patients with chronic PD by conducting a PCR test with 65 chronic PD subjects. Another controlled trial involving 65 patients with aggressive PD and 65 healthy controls discovered that there was no significant difference between the patient and control groups in terms of serum IgG against HSV-1 (76.1 % versus 73.9 %), EBV (98.5 % versus 96.9 %), CMV (47.7 % versus 46.2 %), or IgM levels against HSV-1 (6.2 % versus 1.5 %), EBV (0 % versus 0 %), or CMV (0 % versus 1.5 %) [70].

The discrepancies in the relationship between herpesvirus infection, *H. pylori* infection, and PD have numerous plausible causes. Firstly, in these observational epidemiological studies, there is no prospective, randomized, or blind approach. Biases related to reverse causality, selection bias, and inappropriate confounding control can all affect the differences in results. For herpesvirus, the majority of research that find a positive association with PD use sample sizes of less than thirty, which restricts the generalizability of the findings [70]. Secondly, different studies used different methodologies to diagnose *H. pylori* infection and herpesvirus infection. The World Gastroenterology Organization (WGO) has endorsed urine breath testing as the gold standard for non-invasive methods of detecting *H. pylori* [71]. Nevertheless, the diagnostic standards for *H. pylori* infection vary throughout research since some measure infection with *H. pylori* with blood, anti-*H. pylori* IgG or DNA sequences of *H. pylori*. For herpesviruses, the majority of prior assays are nested PCRs. Despite the fact that this approach is more sensitive than PCR, cross-contamination could lead to false positive results [72]. Thirdly, changes in age, sex, region, ethnicity, dietary habits, and socioeconomic status, as well as the titer of *H. pylori* and herpesvirus IgG, and the type and level of *H. pylori* and herpesvirus antibodies, all contributed to differences in results [[73], [74], [75]]. For instance, the prevalence of *H. pylori* is typically greater than 50 % in communities in Southern and Eastern Europe, South America, and Asia, but roughly one-third of people in Northern and North American populations are still infected [76]. Germany has a high seropositivity rate for HSV-1, ranging from 85.2 % (west) to 88.5 % (east). Furthermore, in Germany, the HSV-1 seroprevalence rate for youth is over 60 %, the rate for individuals between the ages of 35 and 44 is over 80 %, and the rate for the elderly is around 100 % [75]. A survey of CMV prevalence in the United States showed that age (36 % in childhood, 91 % in people aged 80) and race (51 % for non-Hispanics, 76 % for non-Hispanic blacks, and 82 % for Mexican Americans) had a significant impact [77]. The prevalence of EBV in the 30–40 year age group in Western and Central Europe is close to 90 %, while the positive rate in the Central African population is close to 100 %, and the seroprevalence in the United States depends on socioeconomic status [78]. Finally, the characteristic incubation period of herpesvirus can also lead to differences in the detection rate of herpesvirus [79,80].

MR analysis can generate reasonably accurate causal assessments by employing genetic IVs, thus circumventing the impact of these confounding factors [27,81]. Our study has several benefits. Firstly, this is the first research to examine the bidirectional causation between infection with *H. pylori,* herpesvirus and PD using the MR method. This approach can minimize the effects of confounding variables and reverse causality. Secondly, we used multiple GAWS data on subtypes of *H. pylori* and herpesvirus infections to further strengthen the robustness of the conclusions we obtained. Nevertheless, there are some restrictions on our investigation. Initially, individuals with European ancestry are the only subjects of our investigation. It cannot be demonstrated that this is applicable to other racial populations, despite the fact that it would lessen the bias brought about by population stratification. Furthermore, as previously stated, there are racial and geographic differences in the prevalence of *H. pylori* and herpesvirus in patients with PD; therefore, the census population's racial and geographic distributions must be expanded. Secondly, IVs chose a 5e-06 *p*-value threshold, which might somewhat influence the final estimate due to instrumental bias. Thirdly, due to the limited sample size of *H. pylori* and herpesvirus infection-associated GWAS, higher sample sizes will need to be studied in future studies to support the MR analysis. Finally, it is unknown whether study subjects with GWAS data on *H. pylori*, herpesvirus, and PD have used antibiotics now or in the past year, which may affect the readability of our results. The use of antibiotics may omit the detection of patients with *H. pylori* infection and patients with PD. Furthermore, using antibiotics may cause resistance to arise, which may result in genetic loci changing. For instance, mutations in the 23S rRNA, rdxA, frxA, fdxB, gyrA and gyrB loci allow *H. pylori* to avoid the associated antibiotic activity by preventing the synthesis of bacterial proteins, preventing the intracellular reductive activation of metronidazole (MTZ), and preventing the transcription and replication of bacterial DNA, respectively [82,83].

## 5 Conclusions

Taken together, our MR analysis failed to find a causal effect of *H. pylori* and herpesvirus infection on PD or vice versa. This implies that eradicating or preventing *H. pylori* and herpesvirus may not be beneficial for PD. Nevertheless, due to restrictions related to geography and ethnicity as well as the modest size of the GWAS sample size data, the results we obtained should be interpreted cautiously.

## Ethics statement

The data of this study were obtained from public databases, having obtained informed consent from all participating studies in accordance with the protocols approved by their institute's ethics committee. No separate ethical approval is required for this study.

## Funding

This work was supported by the 10.13039/501100001809National Natural Science Foundation of China (82071770); Research Level Improvement Project of 10.13039/501100002947Anhui Medical University (2021xkjT001); 10.13039/501100003995Anhui Provincial Natural Science Foundation (2008085QH371); Scientific Research of BSKY in 10.13039/501100002947Anhui Medical University (XJ201601); Research and practical innovation projects of 10.13039/501100002947AHMU (YJS20230039); Basic and Clinical Cooperative Research and Promotion Program of 10.13039/501100002947Anhui Medical University (2021xkjt039); 10.13039/501100003995Natural Science Foundation of Anhui Province (2208085QH245) and the 10.13039/501100001809National Natural Science Foundation of China (82201127).

## Data availability statement

The GWAS data related to PD were sourced from the GLIDE consortium at https://data.bris.ac.uk/data/dataset/2j2rqgzedxlq02oqbb4vmycnc2. The summary-level data for H. pylori infection, VacA and CagA were obtained from the EBI consortium, available at https://www.ebi.ac.uk/gwas/home/. The FinnGen website provided GWAS data on EBV, CMV and HSV infections. The GWAS data on antibody IgG of EBNA1, VCA, CMV, HSV-1 and HSV-2 were sourced from the Milieu Intérieur cohort at https://doi.org/10.5281/zenodo.1217136.

## CRediT authorship contribution statement

**Erli Wu:** Writing – original draft. **Ming Cheng:** Project administration. **Shouxiang Yang:** Data curation. **Wanting Yuan:** Data curation. **Mengyun Gu:** Software. **Dandan Lu:** Methodology. **Lei Zhang:** Investigation. **Qingqing Wang:** Funding acquisition. **Xiaoyu Sun:** Formal analysis. **Wei Shao:** Writing – review & editing.

## Declaration of competing interest

The authors declare that they have no known competing financial interests or personal relationships that could have appeared to influence the work reported in this paper.

## References

[1] Genco R.J., Sanz M. (2000. 2020). Clinical and public health implications of periodontal and systemic diseases: an overview. Periodontol.

[2] Kinane D.F., Stathopoulou P.G., Papapanou P.N. (2017). Periodontal diseases. Nat. Rev. Dis. Prim..

[3] Ma C. (2023). Periodontitis and stroke: a Mendelian randomization study. Brain Behav.

[4] Marouf N. (2021). Association between periodontitis and severity of COVID-19 infection: a case-control study. J. Clin. Periodontol..

[5] Darveau R.P. (2010). Periodontitis: a polymicrobial disruption of host homeostasis. Nat. Rev. Microbiol..

[6] Hajishengallis G. (2015). Periodontitis: from microbial immune subversion to systemic inflammation. Nat. Rev. Immunol..

[7] Teles F. (2000. 2022). Viruses, periodontitis, and comorbidities. Periodontol.

[8] Chen C., Feng P., Slots J. (2000. 2020). Herpesvirus-bacteria synergistic interaction in periodontitis. Periodontol.

[9] Adachi K. (2019). Influence of Helicobacter pylori infection on periodontitis. J. Gastroenterol. Hepatol..

[10] Liu Y. (2022). No evidence for a causal link between Helicobacter pylori infection and nonalcoholic fatty liver disease: a bidirectional Mendelian randomization study. Front. Microbiol..

[11] Wei X. (2019). The association between chronic periodontitis and oral Helicobacter pylori: a meta-analysis. PLoS One.

[12] Sung C.E. (2022). Periodontitis, Helicobacter pylori infection, and gastrointestinal tract cancer mortality. J. Clin. Periodontol..

[13] Soto C. (2022). Porphyromonas gingivalis-Helicobacter pylori co-incubation enhances Porphyromonas gingivalis virulence and increases migration of infected human oral keratinocytes. J. Oral Microbiol..

[14] Hu Z. (2016). Effect of Helicobacter pylori infection on chronic periodontitis by the change of microecology and inflammation. Oncotarget.

[15] Riggio M.P., Lennon A. (1999). Identification by PCR of Helicobacter pylori in subgingival plaque of adult periodontitis patients. J. Med. Microbiol..

[16] Diouf A. (2011). [Prevalence of Helicobacter pylori detected by real-time PCR in the subgingival plaque of patients with chronic periodontitis]. Odonto-Stomatol. Trop..

[17] Gebara E.C. (2004). Prevalence of Helicobacter pylori detected by polymerase chain reaction in the oral cavity of periodontitis patients. Oral Microbiol. Immunol..

[18] Contreras A., Slots J. (2000). Herpesviruses in human periodontal disease. J. Periodontal. Res..

[19] Nibali L. (2009). Low prevalence of subgingival viruses in periodontitis patients. J. Clin. Periodontol..

[20] Rotola A. (2008). Human herpesvirus 7, Epstein-Barr virus and human cytomegalovirus in periodontal tissues of periodontally diseased and healthy subjects. J. Clin. Periodontol..

[21] Grenier G., Gagnon G., Grenier D. (2009). Detection of herpetic viruses in gingival crevicular fluid of patients suffering from periodontal diseases: prevalence and effect of treatment. Oral Microbiol. Immunol..

[22] Imbronito A.V. (2008). Detection of herpesviruses and periodontal pathogens in subgingival plaque of patients with chronic periodontitis, generalized aggressive periodontitis, or gingivitis. J. Periodontol..

[23] Klemenc P. (2005). Prevalence of some herpesviruses in gingival crevicular fluid. J. Clin. Virol..

[24] Ding F. (2010). [Effect of periodontal mechanical treatment on herpesviruses in gingival crevicular fluid of patients with chronic periodontitis]. Zhonghua Kou Qiang Yi Xue Za Zhi.

[25] Meuli L., Dick F. (2018). Understanding confounding in observational studies. Eur. J. Vasc. Endovasc. Surg..

[26] Anand P.S., Nandakumar K., Shenoy K.T. (2006). Are dental plaque, poor oral hygiene, and periodontal disease associated with Helicobacter pylori infection?. J. Periodontol..

[27] Emdin C.A., Khera A.V., Kathiresan S. (2017). Mendelian randomization. JAMA.

[28] Davies N.M., Holmes M.V., Davey Smith G. (2018). Reading Mendelian randomisation studies: a guide, glossary, and checklist for clinicians. Bmj.

[29] Burgess S., Labrecque J.A. (2018). Mendelian randomization with a binary exposure variable: interpretation and presentation of causal estimates. Eur. J. Epidemiol..

[30] Shungin D. (2019). Genome-wide analysis of dental caries and periodontitis combining clinical and self-reported data. Nat. Commun..

[31] Scepanovic P. (2018). Human genetic variants and age are the strongest predictors of humoral immune responses to common pathogens and vaccines. Genome Med..

[32] Mayerle J. (2013). Identification of genetic loci associated with Helicobacter pylori serologic status. JAMA.

[33] Auton A. (2015). A global reference for human genetic variation. Nature.

[34] Burgess S., Thompson S.G. (2011). Avoiding bias from weak instruments in Mendelian randomization studies. Int. J. Epidemiol..

[35] Pierce B.L., Ahsan H., Vanderweele T.J. (2011). Power and instrument strength requirements for Mendelian randomization studies using multiple genetic variants. Int. J. Epidemiol..

[36] Kamat M.A. (2019). PhenoScanner V2: an expanded tool for searching human genotype-phenotype associations. Bioinformatics.

[37] Staley J.R. (2016). PhenoScanner: a database of human genotype-phenotype associations. Bioinformatics.

[38] Lukkunaprasit T. (2021). Causal associations of urate with cardiovascular risk factors: two-sample mendelian randomization. Front. Genet..

[39] Rasooly D., Patel C.J. (2019). Conducting a reproducible mendelian randomization analysis using the R analytic statistical environment. Curr Protoc Hum Genet.

[40] Lin Z., Deng Y., Pan W. (2021). Combining the strengths of inverse-variance weighting and Egger regression in Mendelian randomization using a mixture of regressions model. PLoS Genet..

[41] Verbanck M. (2018). Publisher Correction: detection of widespread horizontal pleiotropy in causal relationships inferred from Mendelian randomization between complex traits and diseases. Nat. Genet..

[42] Xu W. (2022). Causal association of epigenetic aging and COVID-19 severity and susceptibility: a bidirectional Mendelian randomization study. Front. Med..

[43] Bowden J., Davey Smith G., Burgess S. (2015). Mendelian randomization with invalid instruments: effect estimation and bias detection through Egger regression. Int. J. Epidemiol..

[44] Burgess S., Thompson S.G. (2017). Interpreting findings from Mendelian randomization using the MR-Egger method. Eur. J. Epidemiol..

[45] Verbanck M. (2018). Detection of widespread horizontal pleiotropy in causal relationships inferred from Mendelian randomization between complex traits and diseases. Nat. Genet..

[46] Nolte I.M. (2020). Metasubtract: an R-package to analytically produce leave-one-out meta-analysis GWAS summary statistics. Bioinformatics.

[47] Liu Y. (2020). Periodontal disease and Helicobacter pylori infection in oral cavity: a meta-analysis of 2727 participants mainly based on Asian studies. Clin. Oral Invest..

[48] Valadan Tahbaz S. (2017). Occurrence of Helicobacter pylori and its major virulence genotypes in dental plaque samples of patients with chronic periodontitis in Iran. Gastroenterol Hepatol Bed Bench..

[49] Zou Q.H., Li R.Q. (2011). Helicobacter pylori in the oral cavity and gastric mucosa: a meta-analysis. J. Oral Pathol. Med..

[50] Medina M.L. (2010). Molecular detection of Helicobacter pylori in oral samples from patients suffering digestive pathologies. Med. Oral Patol. Oral Cir. Bucal.

[51] Lauritano D. (2015). Periodontal pockets as a reservoir of HELICOBACTER pylori causing relapse of gastric ulcer: a review of the literature. J. Biol. Regul. Homeost. Agents.

[52] Anand P.S., Kamath K.P., Anil S. (2014). Role of dental plaque, saliva and periodontal disease in Helicobacter pylori infection. World J. Gastroenterol..

[53] Wang Y. (2024). Association of periodontitis with gastrointestinal tract disorders: a bidirectional Mendelian randomization study. J. Periodontol..

[54] Zhang L. (2022). Helicobacter pylori in the oral cavity: current evidence and potential survival strategies. Int. J. Mol. Sci..

[55] Li R. (2023). Association between the presence and genotype of Helicobacter pylori and periodontitis. Exp. Ther. Med..

[56] Al Asqah M. (2009). Is the presence of Helicobacter pylori in dental plaque of patients with chronic periodontitis a risk factor for gastric infection?. Can. J. Gastroenterol..

[57] Moradi Y. (2023). The association between periodontal diseases and helicobacter pylori: an updated meta-analysis of observational studies. BMC Oral Health.

[58] Zaric S. (2009). Periodontal therapy improves gastric Helicobacter pylori eradication. J. Dent. Res..

[59] Ozturk A. (2021). Periodontal treatment is associated with improvement in gastric Helicobacter pylori eradication: an updated meta-analysis of clinical trials. Int. Dent. J..

[60] Salehi M.R. (2013). A comparison in prevalence of Helicobacter pylori in the gingival crevicular fluid from subjects with periodontitis and healthy individuals using polymerase chain reaction. J. Dent. Res. Dent. Clin. Dent. Prospects.

[61] Tsimpiris A. (2023). Association of chronic periodontitis with Helicobacter pylori infection in stomach or mouth: a systematic review and meta-analysis. Eur J Dent.

[62] Shimoyama T. (2013). Helicobacter pylori infection is associated with a decreased risk of tooth loss in healthy Japanese men. Jpn. J. Infect. Dis..

[63] Slots J., Slots H. (2000. 2019). Periodontal herpesvirus morbidity and treatment. Periodontol.

[64] Contreras A., Nowzari H., Slots J. (2000). Herpesviruses in periodontal pocket and gingival tissue specimens. Oral Microbiol. Immunol..

[65] Shah R., Mehta D.S. (2016). Prevalence of herpesviruses in gingivitis and chronic periodontitis: relationship to clinical parameters and effect of treatment. J. Indian Soc. Periodontol..

[66] Kazi M., Bharadwaj R. (2017). Role of herpesviruses in chronic periodontitis and their association with clinical parameters and in increasing severity of the disease. Eur J Dent.

[67] Kubar A. (2005). Real-time polymerase chain reaction quantification of human cytomegalovirus and Epstein-Barr virus in periodontal pockets and the adjacent gingiva of periodontitis lesions. J. Periodontal. Res..

[68] Puletic M. (2020). Detection rates of periodontal bacteria and herpesviruses in different forms of periodontal disease. Microbiol. Immunol..

[69] Dawson D.R. (2009). Real-time polymerase chain reaction to determine the prevalence and copy number of epstein-barr virus and cytomegalovirus DNA in subgingival plaque at individual healthy and periodontal disease sites. J. Periodontol..

[70] Stein J.M. (2013). Failure to detect an association between aggressive periodontitis and the prevalence of herpesviruses. J. Clin. Periodontol..

[71] Katelaris P. (2023). Helicobacter pylori world Gastroenterology organization global guideline. J. Clin. Gastroenterol..

[72] Botero J.E. (2008). Comparison of nested polymerase chain reaction (PCR), real-time PCR and viral culture for the detection of cytomegalovirus in subgingival samples. Oral Microbiol. Immunol..

[73] Nahmias A.J., Lee F.K., Beckman-Nahmias S. (1990). Sero-epidemiological and -sociological patterns of herpes simplex virus infection in the world. Scand. J. Infect. Dis. Suppl..

[74] Pebody R.G. (2004). The seroepidemiology of herpes simplex virus type 1 and 2 in Europe. Sex. Transm. Infect..

[75] Hellenbrand W. (2005). Seroprevalence of herpes simplex virus type 1 (HSV-1) and type 2 (HSV-2) in former East and West Germany, 1997-1998. Eur. J. Clin. Microbiol. Infect. Dis..

[76] Eusebi L.H., Zagari R.M., Bazzoli F. (2014). Epidemiology of Helicobacter pylori infection. Helicobacter.

[77] Staras S.A. (2006). Seroprevalence of cytomegalovirus infection in the United States, 1988-1994. Clin. Infect. Dis..

[78] Vetsika E.K., Callan M. (2004). Infectious mononucleosis and Epstein-Barr virus. Expert Rev Mol Med.

[79] Arora N. (2010). Cytomegalovirus viruria and DNAemia in healthy seropositive women. J. Infect. Dis..

[80] Scheithauer S. (2010). Impact of herpes simplex virus detection in respiratory specimens of patients with suspected viral pneumonia. Infection.

[81] Sheehan N.A. (2008). Mendelian randomisation and causal inference in observational epidemiology. PLoS Med..

[82] Gong Y., Yuan Y. (2018). Resistance mechanisms of Helicobacter pylori and its dual target precise therapy. Crit. Rev. Microbiol..

[83] Alba C., Blanco A., Alarcón T. (2017). Antibiotic resistance in Helicobacter pylori. Curr. Opin. Infect. Dis..

