# Supplementary material for: Causal relationships of infection with Helicobacter pylori and herpesvirus on periodontitis: A Mendelian randomization study
Source: Heliyon. 2024 Aug 6;10(16):e35904. doi: 10.1016/j.heliyon.2024.e35904 (PMC11365429; doi:10.1016/j.heliyon.2024.e35904)
Supplement: Multimedia component 1 [file mmc1.docx]

**Table S1** Instrument variants of *H. pylori* infection and *F*-statistic.

| **SNP** | **Gene** | **EA** | **OA** | **Beta** | **se** | **P-value** | **EAF** | ***F*-statistic** |
| --- | --- | --- | --- | --- | --- | --- | --- | --- |
| rs10004195 | TLR1 | A | T | -0.36 | 0.04 | 1.40E-18 | 0.25 | 558.639 |
| rs368433 | FCGR2A | C | T | 0.31 | 0.06 | 2.10E-08 | 0.16 | 289.986 |

Abbreviations: SNPs, single-nucleotide polymorphisms; EA, effect allele; OA, other allele; EAF, effect allele frequency.

**Table S2** Detailed information of instrumental variables used in the Mendelian randomization analysis of VacA on PD (significant level of p < 5e-06).

| **SNP** | **EA** | **OA** | ***F*-statistic** | **associated with VacA** | | | **associated with PD** | | |
| --- | --- | --- | --- | --- | --- | --- | --- | --- | --- |
|  |  |  |  | **Beta** | **se** | **P-value** | **Beta** | **se** | **P-value** |
| rs10246445 | C | A | 20.80041925 | -0.488 | 0.107 | 4.920E-06 | 0.026 | 0.053 | 0.625 |
| rs113845906 | A | G | 23.60816609 | 0.413 | 0.085 | 9.974E-07 | 0.054 | 0.050 | 0.274 |
| rs117077218 | T | C | 21.19973086 | 0.640 | 0.139 | 3.949E-06 | -0.034 | 0.088 | 0.694 |
| rs133537 | C | T | 22.11541271 | -0.174 | 0.037 | 2.252E-06 | 0.035 | 0.018 | 0.046 |
| rs1530121 | C | T | 27.20338331 | -0.266 | 0.051 | 1.956E-07 | -0.019 | 0.022 | 0.400 |
| rs7019543 | T | G | 22.68767313 | 0.181 | 0.038 | 2.008E-06 | -0.031 | 0.016 | 0.063 |
| rs72645538 | G | A | 21.08153629 | 0.753 | 0.164 | 4.657E-06 | -0.185 | 0.157 | 0.241 |
| rs77497849 | G | A | 23.90123457 | 0.264 | 0.054 | 9.129E-07 | 0.024 | 0.024 | 0.316 |
| rs9606224 | T | C | 24.71649462 | 0.5071 | 0.102 | 6.909E-07 | 0.005 | 0.054 | 0.927 |
| rs372744619 | C | A | 25.98655701 | 0.887 | 0.174 | 3.290E-07 | NA | NA | NA |

Abbreiations: SNPs, single-nucleotide polymorphisms; IVW, inverse variance-weighted method; *H. pylori*, *Helicobacter pylori*; VacA, Vacuolar cytotoxin A; PD, periodontitis; EA, effect allele; OA, other allele.

**Table S3** Detailed information of instrumental variables used in the Mendelian randomization analysis of CacA on PD (significant level of p < 5e-06)

| **SNP** | **EA** | **OA** | ***F*-statistic** | **associated with CagA** | | | **associated with PD** | | |
| --- | --- | --- | --- | --- | --- | --- | --- | --- | --- |
|  |  |  |  | **Beta** | **se** | **P-value** | **Beta** | **se** | **P-value** |
| rs117827497 | G | A | 21.827584 | -0.584 | 0.125 | 3.096E-06 | -0.045 | 0.055 | 0.416 |
| rs118006294 | C | T | 21.68637063 | -0.475 | 0.102 | 3.038E-06 | -0.014 | 0.082 | 0.868 |
| rs11858369 | G | A | 25.93888588 | 0.438 | 0.086 | 4.182E-07 | 0.111 | 0.041 | 0.007 |
| rs138363822 | G | A | 20.62673611 | 0.218 | 0.048 | 4.304E-06 | 0.017 | 0.021 | 0.420 |
| rs4268452 | T | C | 21.00694444 | -0.385 | 0.084 | 4.622E-06 | -0.024 | 0.038 | 0.532 |
| rs75170215 | C | T | 22.68445924 | 0.743 | 0.156 | 2.016E-06 | -0.171 | 0.155 | 0.269 |
| rs75740599 | A | G | 21.1635869 | 0.236 | 0.0513 | 4.273E-06 | -0.003 | 0.019 | 0.865 |

Abbreviations: SNPs, single-nucleotide polymorphisms; CagA, Cytotoxin-associated protein A; PD, periodontitis; EA, effect allele; OA, other allele.

**Table S4** Detailed information of instrumental variables used in the Mendelian randomization analysis of PD on *H. pylori* infection, VacA, and CacA (significant level of p < 5e-06).

| **SNP** | **EA** | **OA** | ***F*-statistic** | **associated with PD** | | | **associated with *H. pylori* infection** | | | **associated with VacA** | | | **associated with CagA** | | |
| --- | --- | --- | --- | --- | --- | --- | --- | --- | --- | --- | --- | --- | --- | --- | --- |
|  |  |  |  | **Beta** | **se** | **P-value** | **Beta** | **se** | **P-value** | **Beta** | **se** | **pval** | **Beta** | **se** | **P-value** |
| rs10143801 | G | A | 24.13050169 | -0.084 | 0.0171 | 8.66E-07 | 0.043 | 0.027 | 0.107 | 0.006 | 0.040 | 0.889 | -0.014 | 0.050 | 0.772 |
| rs151226594 | G | T | 22.84790209 | -0.3671 | 0.0768 | 1.75E-06 | -0.015 | 0.0939 | 0.877 | 0.082 | 0.150 | 0.583 | 0.057 | 0.174 | 0.744 |
| rs73155039 | G | A | 22.40193013 | 0.8316 | 0.1757 | 2.22E-06 | 0.169 | 0.105 | 0.108 | -0.068 | 0.161 | 0.673 | 0.176 | 0.185 | 0.341 |
| rs76734229 | A | G | 22.65245435 | -0.1761 | 0.037 | 1.94E-06 | 0.081 | 0.042 | 0.053 | -0.020 | 0.063 | 0.757 | -0.002 | 0.078 | 0.974 |
| rs9954920 | T | C | 22.2575558 | 0.0769 | 0.0163 | 2.37E-06 | 0.021 | 0.024 | 0.372 | -0.002 | 0.037 | 0.957 | 0.029 | 0.046 | 0.539 |

Abbreviations: SNPs, single-nucleotide polymorphisms; VacA, Vacuolar cytotoxin A; CagA, Cytotoxin-associated protein A; PD, periodontitis; EA, effect allele; OA, other allele.

**Table S5** Detailed information of instrumental variables used in the Mendelian randomization analysis of herpesvirus infection on PD (significant level of p < 5e-06).

| **Exposure** | **SNP** | **EA** | **OA** | ***F*-statistic** | **Beta** | **se** | **P-value** | **PD** | | |
| --- | --- | --- | --- | --- | --- | --- | --- | --- | --- | --- |
|  |  |  |  |  |  |  |  | **Beta** | **se** | **P-value** |
| EBV infection | rs10468923 | C | T | 25.66524677 | -0.14239 | 0.0281065 | 4.06E-07 | -0.0245 | 0.0187 | 0.19 |
| EBV infection | rs112339046 | A | G | 23.6673029 | 0.390882 | 0.0803473 | 1.15E-06 | 0.1047 | 0.1301 | 0.4209 |
| EBV infection | rs12358176 | T | C | 21.11795183 | -0.124696 | 0.0271348 | 4.32E-06 | -0.0269 | 0.0183 | 0.141 |
| EBV infection | rs12598357 | G | A | 27.42620716 | 0.136845 | 0.0261304 | 1.63E-07 | -0.0227 | 0.0242 | 0.3479 |
| EBV infection | rs2618374 | T | C | 21.64449982 | 0.132658 | 0.0285141 | 3.28E-06 | -0.0328 | 0.019 | 0.08412 |
| EBV infection | rs28529232 | G | A | 21.6843266 | 0.223307 | 0.0479545 | 3.21E-06 | -0.0419 | 0.0228 | 0.06567 |
| EBV infection | rs3130169 | T | C | 24.17553525 | 0.180085 | 0.036626 | 8.79E-07 | -0.0016 | 0.0396 | 0.9674 |
| EBV infection | rs318497 | A | G | 47.51747881 | 0.183922 | 0.0266813 | 5.45E-12 | 0.0289 | 0.0153 | 0.05899 |
| EBV infection | rs56253436 | G | A | 20.9830871 | -0.187465 | 0.0409247 | 4.63E-06 | -0.0435 | 0.0385 | 0.2583 |
| EBV infection | rs59257919 | C | T | 22.49757412 | 0.213345 | 0.0449795 | 2.10E-06 | 0.0192 | 0.0237 | 0.4182 |
| EBV infection | rs76206169 | A | G | 24.20488445 | -1.4323 | 0.291127 | 8.66E-07 | 0.005 | 0.0861 | 0.9538 |
| HSV infection | rs10234639 | G | T | 21.01816752 | 0.120153 | 0.0262082 | 4.55E-06 | -0.0092 | 0.0163 | 0.5737 |
| HSV infection | rs10961236 | A | G | 22.58604232 | 0.125742 | 0.0264582 | 2.01E-06 | -0.006 | 0.0172 | 0.7278 |
| HSV infection | rs12457005 | C | T | 26.74632354 | 0.289393 | 0.0559572 | 2.32E-07 | -0.0025 | 0.033 | 0.9398 |
| HSV infection | rs148444866 | A | G | 21.36583704 | 0.31358 | 0.0678404 | 3.79E-06 | -0.0094 | 0.081 | 0.9074 |
| HSV infection | rs2004786 | T | G | 20.98967704 | -0.114692 | 0.025034 | 4.62E-06 | -0.0025 | 0.0164 | 0.8798 |
| HSV infection | rs4716482 | C | A | 24.55967903 | -0.115749 | 0.0233564 | 7.20E-07 | 0.0132 | 0.0162 | 0.414 |
| HSV infection | rs59142651 | A | G | 21.18862938 | -0.451457 | 0.0980765 | 4.16E-06 | 0.0163 | 0.045 | 0.7171 |
| HSV infection | rs71428759 | G | T | 22.25165502 | 0.323057 | 0.0684854 | 2.39E-06 | 0.0074 | 0.0633 | 0.9069 |
| HSV infection | rs75710096 | G | A | 21.39712766 | 0.234185 | 0.0506269 | 3.73E-06 | 0.0406 | 0.0349 | 0.2438 |
| HSV infection | rs77231357 | G | A | 22.67127771 | 0.227101 | 0.0476959 | 1.92E-06 | -0.0223 | 0.0453 | 0.6222 |
| HSV infection | rs9263969 | T | C | 24.64852584 | -0.136252 | 0.027444 | 6.88E-07 | 0.0472 | 0.0251 | 0.06023 |
| CMV infection | rs11658622 | A | C | 28.52874428 | 0.445347 | 0.0833791 | 9.23E-08 | 0 | 0.0198 | 0.999 |
| CMV infection | rs4349860 | T | C | 24.90456708 | 0.486119 | 0.0974099 | 6.02E-07 | 0.0154 | 0.0282 | 0.5839 |
| CMV infection | rs7760051 | C | T | 23.91842076 | 0.34088 | 0.0697004 | 1.01E-06 | 0.0211 | 0.0203 | 0.298 |
| EBNA1 IgG | rs17452718 | G | T | 21.56282626 | -0.177583 | 0.0382427 | 3.93E-06 | -0.027 | 0.0235 | 0.2503 |
| EBNA1 IgG | rs4555924 | G | A | 23.23108875 | 0.190281 | 0.0394785 | 1.68E-06 | -0.005 | 0.0251 | 0.8409 |
| EBNA1 IgG | rs530411 | T | C | 25.4923971 | 0.139879 | 0.0277043 | 5.37E-07 | 0.0417 | 0.0163 | 0.01055 |
| EBNA1 IgG | rs59217282 | T | C | 22.16584785 | -0.193257 | 0.0410481 | 2.89E-06 | -0.0059 | 0.0264 | 0.8243 |
| EBNA1 IgG | rs6895504 | C | T | 21.46183942 | 0.147586 | 0.0318575 | 4.14E-06 | -0.0067 | 0.019 | 0.7229 |
| EBNA1 IgG | rs6927022 | G | A | 47.62581252 | -0.176483 | 0.025573 | 9.71E-12 | 0.0254 | 0.0249 | 0.3061 |
| VCA IgG | rs2163916 | A | G | 21.16690116 | -0.121204 | 0.0263444 | 4.78E-06 | -0.023 | 0.0209 | 0.2702 |
| VCA IgG | rs245064 | T | C | 21.33470529 | -0.100731 | 0.0218082 | 4.39E-06 | 0.0062 | 0.016 | 0.6994 |
| VCA IgG | rs6556882 | C | T | 24.16379688 | -0.109206 | 0.0222159 | 1.04E-06 | 0.0216 | 0.0163 | 0.1859 |
| VCA IgG | rs6985207 | C | A | 23.46327599 | -0.104443 | 0.0215618 | 1.49E-06 | -0.0244 | 0.0173 | 0.1592 |
| VCA IgG | rs9876198 | T | C | 25.61804101 | -0.108765 | 0.021489 | 5.00E-07 | -0.0115 | 0.016 | 0.4708 |
| HSV-1 IgG | rs10977313 | T | G | 26.90607771 | -0.125265 | 0.0241493 | 2.97E-07 | -0.0239 | 0.0243 | 0.3255 |
| HSV-1 IgG | rs3132935 | G | A | 22.42212669 | 0.0937333 | 0.019795 | 2.76E-06 | 0.0693 | 0.0244 | 0.004427 |
| HSV-1 IgG | rs58599785 | T | C | 21.27720541 | 0.0865176 | 0.0187563 | 4.91E-06 | -0.0156 | 0.0212 | 0.4614 |
| HSV-2 IgG | rs10174926 | C | T | 25.88536785 | -0.236428 | 0.0464699 | 9.72E-07 | -0.0241 | 0.0249 | 0.3325 |
| HSV-2 IgG | rs10782620 | G | T | 23.70171131 | 0.162769 | 0.0334335 | 2.60E-06 | 0.0069 | 0.0158 | 0.6603 |
| HSV-2 IgG | rs10790877 | G | A | 26.37180815 | 0.162333 | 0.0316109 | 7.82E-07 | -7.00E-04 | 0.0154 | 0.9622 |
| HSV-2 IgG | rs10964023 | T | G | 23.0039944 | -0.193637 | 0.0403726 | 3.58E-06 | -0.0187 | 0.0225 | 0.4053 |
| HSV-2 IgG | rs35213774 | G | A | 25.59973164 | 0.265169 | 0.0524089 | 1.10E-06 | 0.0044 | 0.0235 | 0.85 |
| HSV-2 IgG | rs355547 | C | T | 24.28022185 | 0.17259 | 0.0350259 | 2.00E-06 | -0.0159 | 0.0157 | 0.3109 |
| HSV-2 IgG | rs72804080 | G | A | 29.54242429 | 0.259082 | 0.0476666 | 1.92E-07 | 0.0078 | 0.0237 | 0.744 |
| CMV IgG | rs1600519 | T | G | 32.87984401 | -0.255806 | 0.0446114 | 2.20E-08 | -0.0675 | 0.0341 | 0.04775 |
| CMV IgG | rs76825464 | T | C | 25.40343494 | -0.135782 | 0.0269399 | 7.64E-07 | 0.0038 | 0.0218 | 0.8605 |
| CMV IgG | rs79686415 | A | C | 33.87198864 | -0.167317 | 0.0287488 | 1.38E-08 | 0.0658 | 0.0307 | 0.03209 |
| CMV IgG | rs77577412 | A | G | 24.03544254 | -0.241419 | 0.0492431 | 1.48E-06 | -0.0425 | 0.0382 | 0.2667 |
| CMV IgG | rs4899627 | A | C | 21.94710432 | 0.138971 | 0.0296644 | 4.09E-06 | -0.0093 | 0.022 | 0.6733 |
| CMV IgG | rs35701456 | C | A | 30.22204294 | -0.272371 | 0.0495449 | 7.66E-08 | -0.0105 | 0.0369 | 0.7761 |
| CMV IgG | rs72862405 | A | G | 22.00400443 | 0.236069 | 0.0503255 | 3.97E-06 | -0.0174 | 0.0329 | 0.597 |
| CMV IgG | rs76027104 | A | C | 25.49530861 | -0.12739 | 0.0252293 | 7.31E-07 | -0.0299 | 0.0242 | 0.2161 |
| CMV IgG | rs58607100 | A | G | 21.92692808 | -0.184646 | 0.0394322 | 4.13E-06 | -0.0256 | 0.0359 | 0.4756 |
| CMV IgG | rs1738233 | G | A | 27.42056422 | -0.218403 | 0.0417081 | 2.90E-07 | 0.016 | 0.0323 | 0.6213 |
| CMV IgG | rs58599785 | A | C | 23.81418761 | -0.217079 | 0.0444836 | 1.65E-06 | 0.0621 | 0.0409 | 0.1287 |
| CMV IgG | rs10977313 | T | C | 23.86265002 | -0.176426 | 0.0361163 | 1.61E-06 | 0.0599 | 0.0357 | 0.0935 |

Abbreviations: SNP, single-nucleotide polymorphism; EA, effect allele; OA, other allele; EBV, Epstein-Barr virus; CMV, cytomegalovirus; HSV, herpes simplex; EBNA1, Epstein-Barr virus nuclear antigen-1; VCA, EBV viral capsid antigen; HSV-1, HSV type 1; HSV-2, HSV type 2; PD, periodontitis.

**Table S6** Detailed information of instrumental variables used in the Mendelian randomization analysis of PD on herpesvirus infection (significant level of p < 5e-06).

| **SNP** | **EA** | **OA** | ***F*-statistic** | **associated with PD** | | | **associated with EBV** | | | **associated with HSV** | | | **associated with CMV** | | | **associated with EBNA1 IgG** | | | **associated with VCA IgG** | | | **associated with HSV-1 IgG** | | | **associated with HSV-2 IgG** | | | **associated with CMV IgG** | | | |
| --- | --- | --- | --- | --- | --- | --- | --- | --- | --- | --- | --- | --- | --- | --- | --- | --- | --- | --- | --- | --- | --- | --- | --- | --- | --- | --- | --- | --- | --- | --- | --- |
|  |  |  |  | **Beta** | **se** | **P-value** | **Beta** | **se** | **P-value** | **Beta** | **se** | **pval** | **Beta** | **se** | **P-value** | **Beta** | **se** | **P-value** | **Beta** | **se** | **P-value** | **Beta** | **se** | **P-value** | **Beta** | **se** | **P-value** | **Beta** | **se** | **P-value** | |
| rs10143801 | A | G | 24.13050169 | -0.084 | 0.0171 | 8.66E-07 | 0.027328 | 0.0276178 | 0.322416 | 0.00444512 | 0.0245836 | 0.856512 | 0.0506251 | 0.0721728 | 0.483027 | 0.00837137 | 0.0308002 | 0.785841572 | -0.0368871 | 0.0251947 | 0.143503998 | 0.0117517 | 0.0173673 | 0.498895975 | 0.0235348 | 0.0346094 | 0.497443774 | -0.0191743 | 0.0242682 | | 0.430029253 |
| rs138868497 | T | C | 24.30395079 | 1.6387 | 0.3324 | 8.20E-07 | -0.189215 | 0.117696 | 0.107911 | 0.0502181 | 0.104158 | 0.629709 | -0.390117 | 0.300254 | 0.193844 | NA | NA | NA | NA | NA | NA | NA | NA | NA | NA | NA | NA | NA | NA | | NA |
| rs151226594 | T | G | 22.84790209 | -0.3671 | 0.0768 | 1.75E-06 | -0.209254 | 0.091598 | 0.0223429 | -0.0694189 | 0.0820316 | 0.397416 | -0.181277 | 0.23645 | 0.443282 | NA | NA | NA | NA | NA | NA | NA | NA | NA | NA | NA | NA | NA | NA | | NA |
| rs73155039 | A | G | 22.40193013 | 0.8316 | 0.1757 | 2.22E-06 | 0.076321 | 0.179187 | 0.670159 | -0.156306 | 0.158927 | 0.325356 | 0.079774 | 0.438872 | 0.855763 | NA | NA | NA | NA | NA | NA | NA | NA | NA | NA | NA | NA | NA | NA | | NA |
| rs76734229 | A | G | 22.65245435 | -0.1761 | 0.037 | 1.94E-06 | -0.0134723 | 0.0462893 | 0.771017 | 0.0200446 | 0.0411807 | 0.626436 | 0.055901 | 0.120853 | 0.643684 | -0.0168245 | 0.0501126 | 0.737148524 | -0.0126296 | 0.0407091 | 0.756447862 | -0.0353398 | 0.0292771 | 0.227902978 | -0.0191341 | 0.0642767 | 0.766316188 | 0.0502511 | 0.0375021 | | 0.181171967 |
| rs9954920 | T | C | 22.2575558 | 0.0769 | 0.0163 | 2.37E-06 | -0.0520095 | 0.0281184 | 0.0643621 | -0.0047901 | 0.0249966 | 0.848032 | 0.0192442 | 0.0732749 | 0.792835 | 0.0235078 | 0.0275554 | 0.393823838 | -0.0243918 | 0.0224636 | 0.277829271 | 0.0155545 | 0.0156919 | 0.321988232 | 0.00369138 | 0.0345752 | 0.91510494 | NA | NA | | NA |

Abbreviations: SNP, single-nucleotide polymorphism; EA, effect allele; OA, other allele; EBV, Epstein-Barr virus; CMV, cytomegalovirus; HSV, herpes simplex; EBNA1, Epstein-Barr virus nuclear antigen-1; VCA, EBV viral capsid antigen;HSV-1, HSV type 1; HSV-2, HSV type 2; PD, periodontitis.


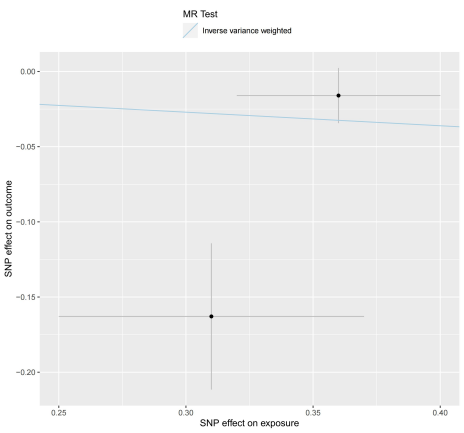

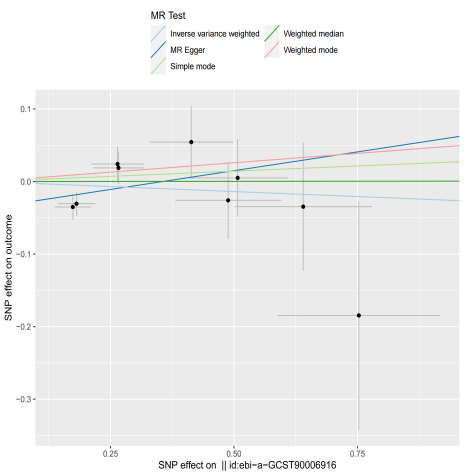

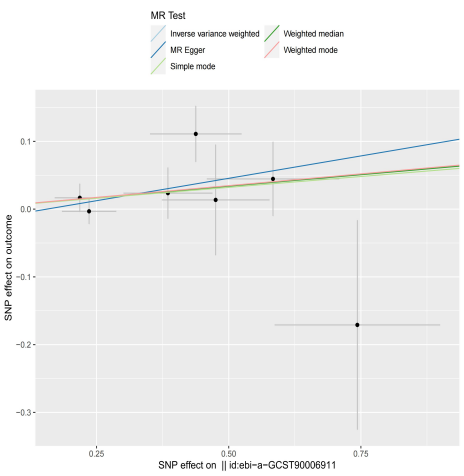


A

B

C


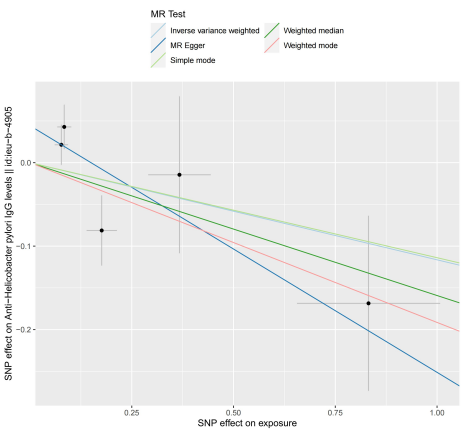

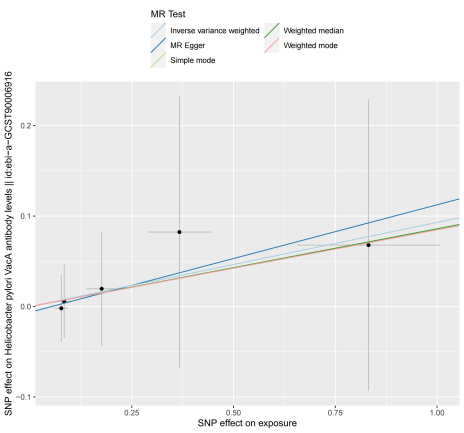

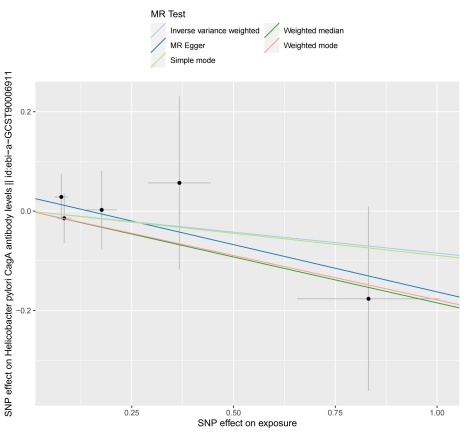


D

E

F

**Fig. S1** Scatter plots of MR between *H. pylori* infection and PD. (A) *H. pylori* on PD; (B) VacA on PD; (C) CagA on PD; (D) PD on *H. pylori*; (E) PD on VacA; (F) PD on CagA; Abbreviations: *H. pylori*, *Helicobacter pylori*; VacA, Vacuolar cytotoxin A; CagA; PD, periodontitis; MR Mendelian randomization.


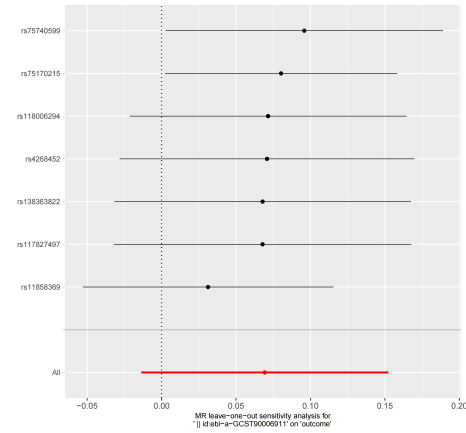

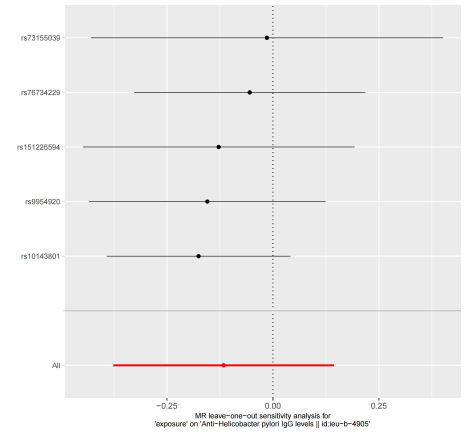

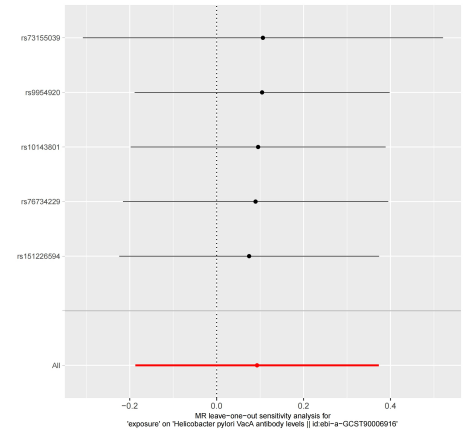

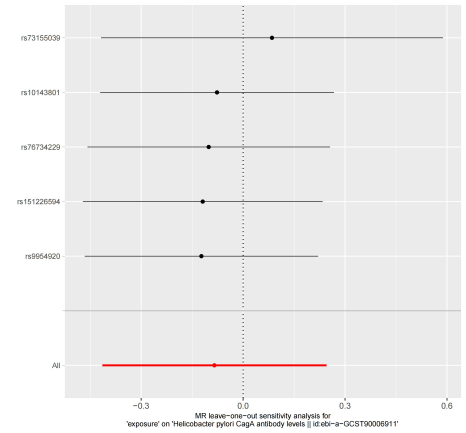


E

B


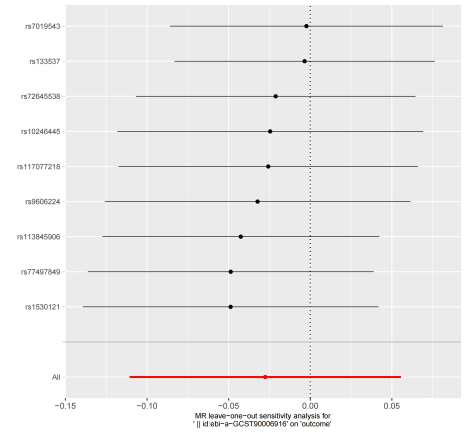


A

C

D

**Fig. S2** Leave-one-out plot of MR analyses. (A) VacA on PD; (B) CagA on PD; (C) PD on *H. pylori*; (D) PD on VacA; (E) PD on CagA. Abbreviations: *H. pylori*, *Helicobacter pylori*; VacA, Vacuolar cytotoxin A; CagA; PD, periodontitis; MR Mendelian randomization.


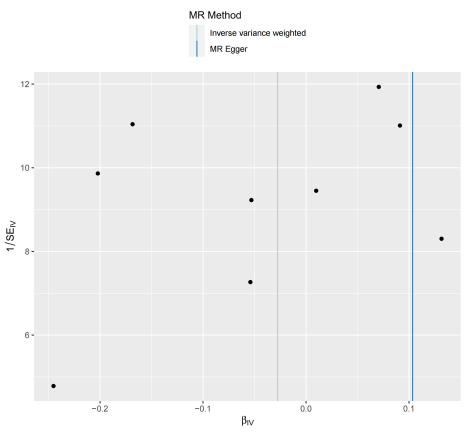

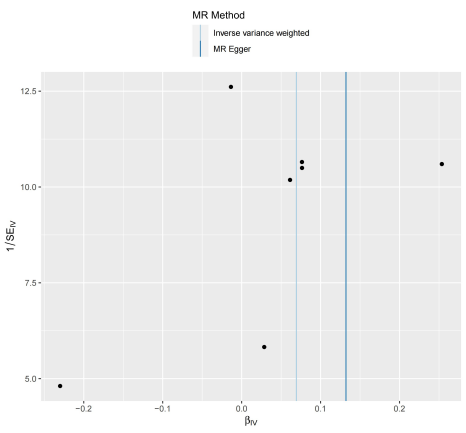

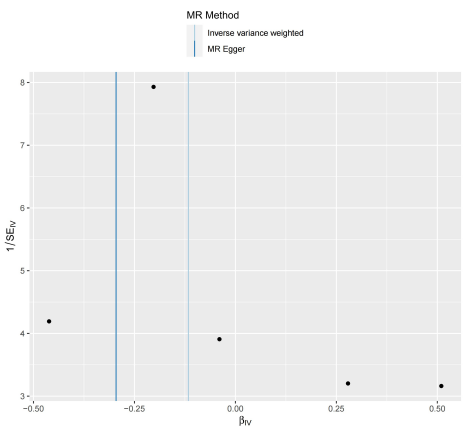

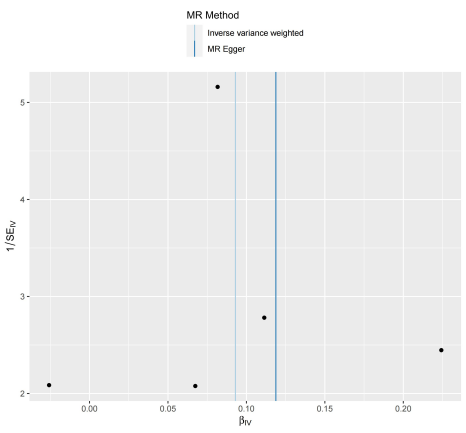

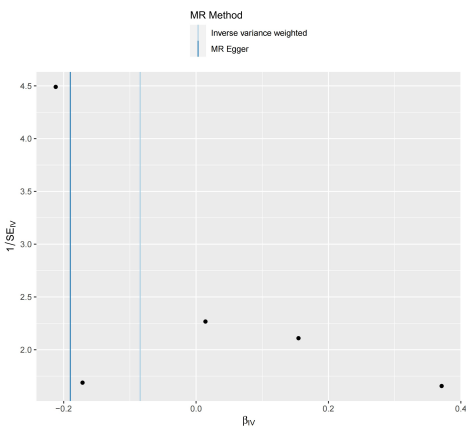


E

B

A

C

D

**Fig. S3** Funnel plot of MR analyses. (A) VacA on PD; (B) CagA on PD; (C) PD on *H. pylori*; (D) PD on VacA; (E) PD on CagA. Abbreviations: *H. pylori*, *Helicobacter pylori*; VacA, Vacuolar cytotoxin A; CagA; PD, periodontitis; MR Mendelian randomization.


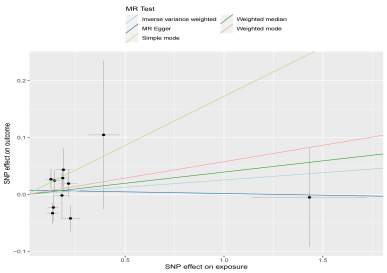

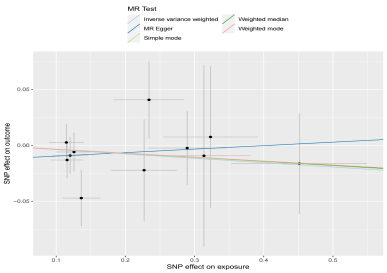

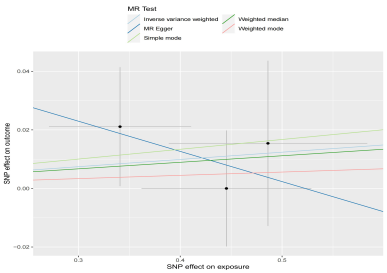

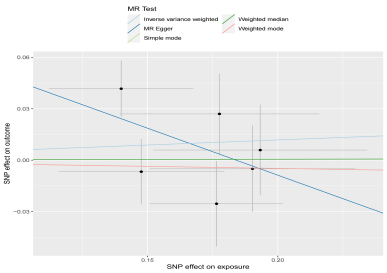

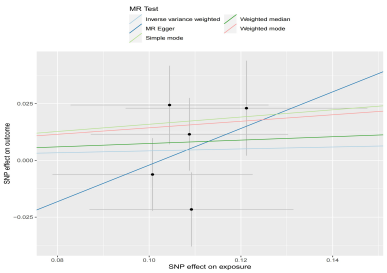

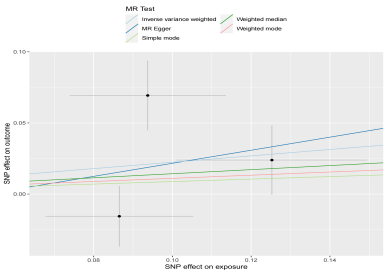

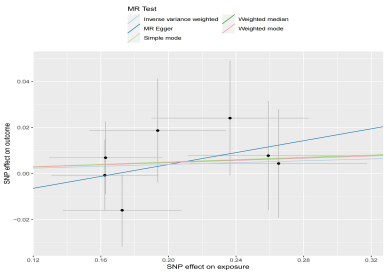

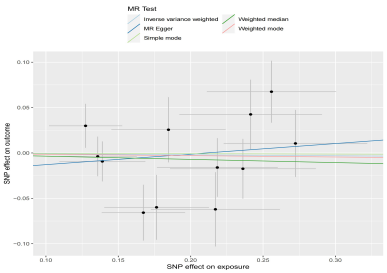


B

A

C

D

F

E

G

H


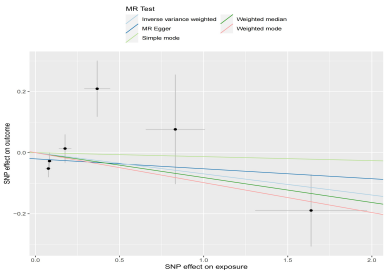

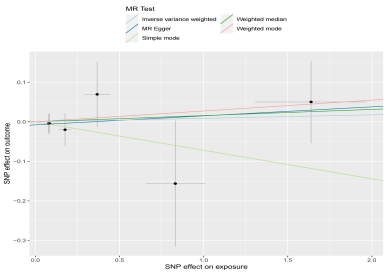

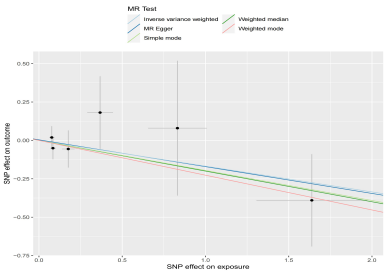

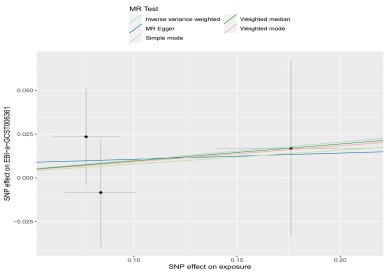

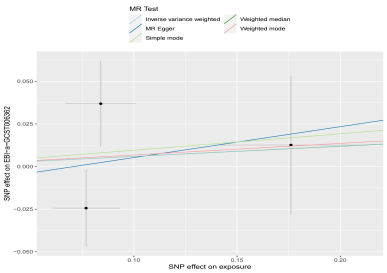

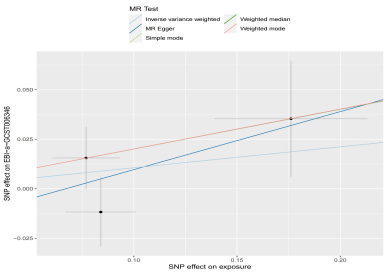

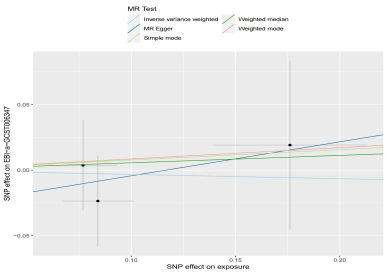

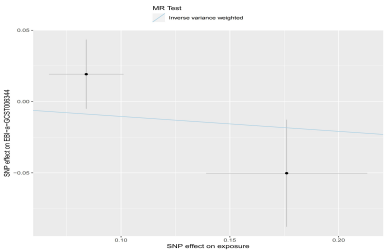


J

I

K

L

N

M

O

P

**Fig. S4** Scatter plots of MR between herpesvirus infection and PD. (A) EBV infection on PD; (B) HSV infection on PD; (C) CMV infection on PD; (D) EBNA1 IgG on PD; (E) VCA IgG on PD; (F) HSV-1 IgG on PD; (G) HSV-2 IgG on PD; (H) CMV IgG on PD; (I) PD on EBV infection; (J) PD on HSV infection; (K) PD on CMV infection; (L) PD on EBNA1 IgG; (M) PD on VCA IgG; (N) PD HSV-1 IgG; (O) PD on HSV-2 IgG; (P) PD on CMV IgG; Abbreviations: EBV, Epstein-Barr virus; CMV, cytomegalovirus; HSV, herpes simplex; EBNA1, Epstein-Barr virus nuclear antigen-1; VCA, EBV viral capsid antigen; HSV-1, HSV type 1; HSV-2, HSV type 2; PD, periodontitis; MR Mendelian randomization.


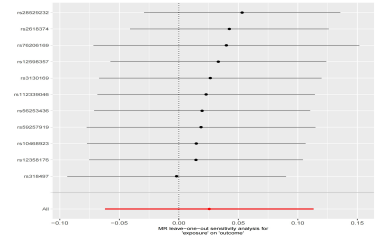

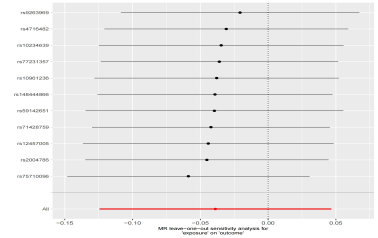

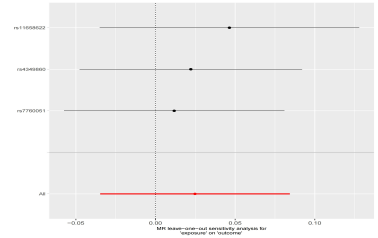

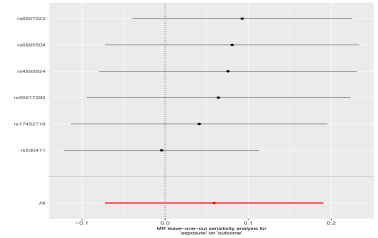

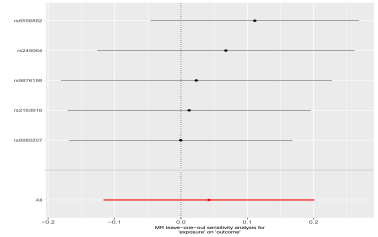

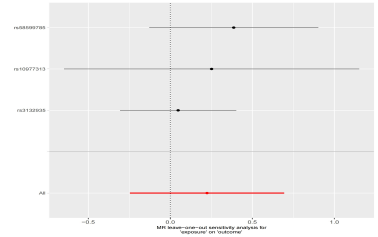

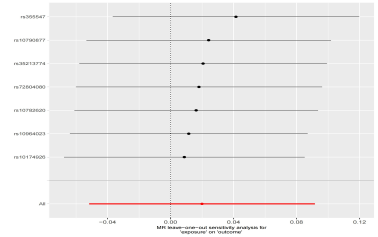

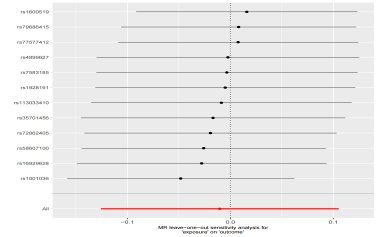


B

A

C

D

F

E

G

H


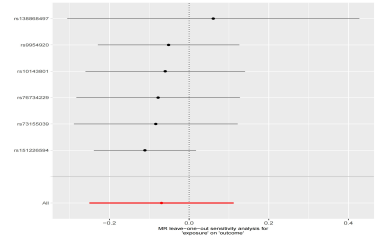

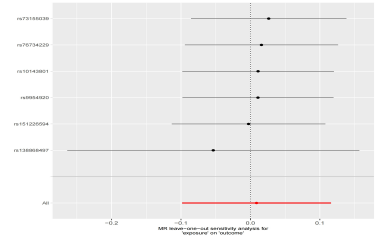

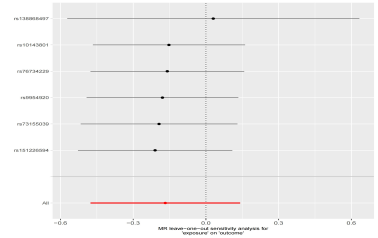

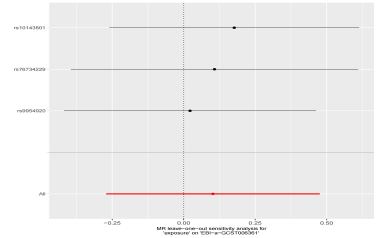

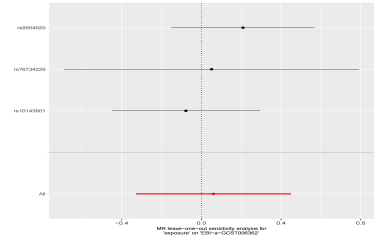

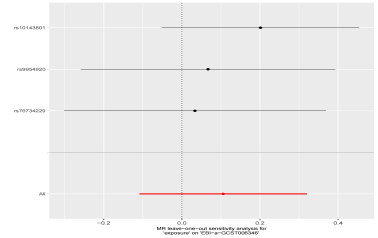

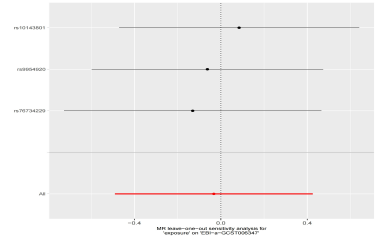


J

I

K

L

N

M

O

**Fig. S5** Leave-one-out plot of MR analyses. (A) EBV infection on PD; (B) HSV infection on PD; (C) CMV infection on PD; (D) EBNA1 IgG on PD; (E) VCA IgG on PD; (F) HSV-1 IgG on PD; (G) HSV-2 IgG on PD; (H) CMV IgG on PD; (I) PD on EBV infection; (J) PD on HSV infection; (K) PD on CMV infection; (L) PD on EBNA1 IgG; (M) PD on VCA IgG; (N) PD HSV-1 IgG; (O) PD on HSV-2 IgG; Abbreviations: EBV, Epstein-Barr virus; CMV, cytomegalovirus; HSV, herpes simplex; EBNA1, Epstein-Barr virus nuclear antigen-1; VCA, EBV viral capsid antigen; HSV-1, HSV type 1; HSV-2, HSV type 2; PD, periodontitis; MR Mendelian randomization.


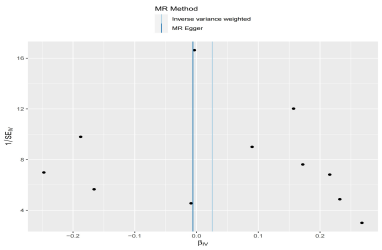

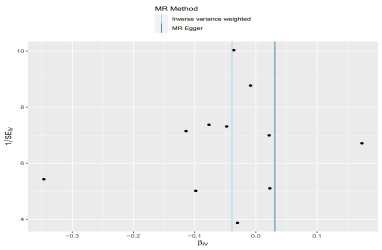

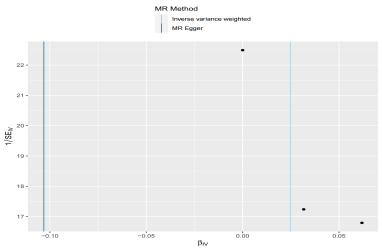

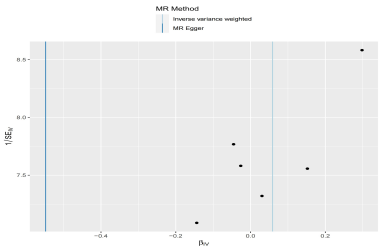

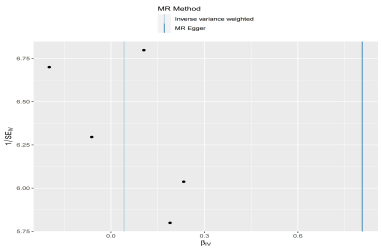

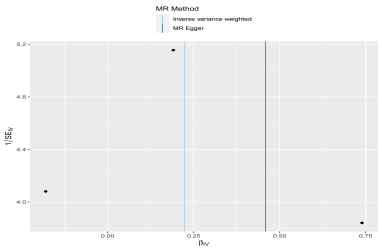

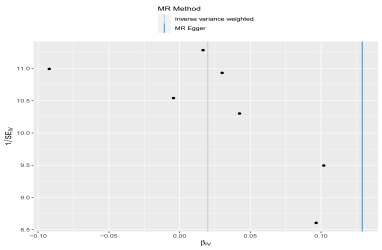

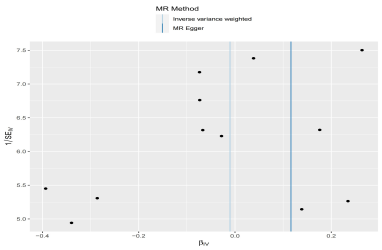


B

A

C

D

F

E

G

H


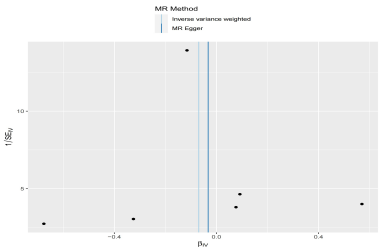

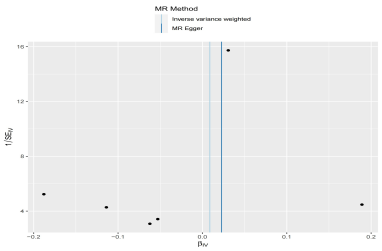

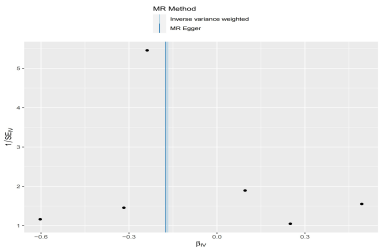

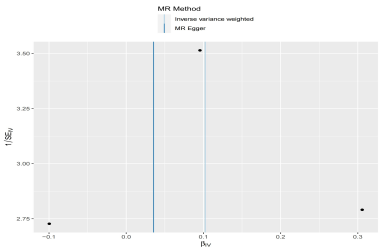

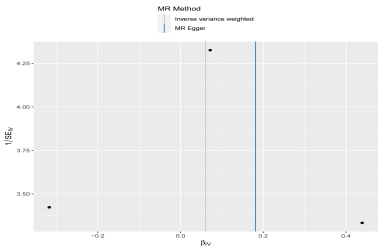

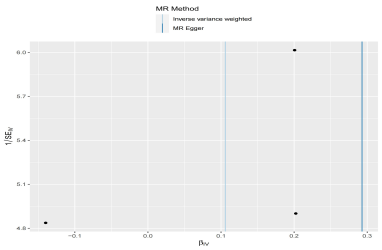

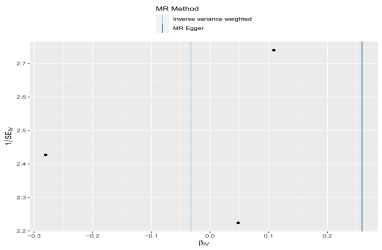

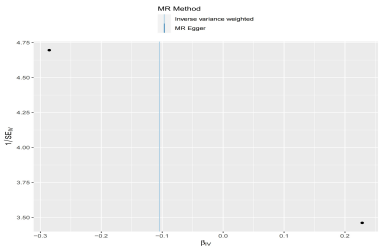


J

I

K

L

N

M

O

P

**Fig. S6** Funnel plot of MR analyses. (A) EBV infection on PD; (B) HSV infection on PD; (C) CMV infection on PD; (D) EBNA1 IgG on PD; (E) VCA IgG on PD; (F) HSV-1 IgG on PD; (G) HSV-2 IgG on PD; (H) CMV IgG on PD; (I) PD on EBV infection; (J) PD on HSV infection; (K) PD on CMV infection; (L) PD on EBNA1 IgG; (M) PD on VCA IgG; (N) PD HSV-1 IgG; (O) PD on HSV-2 IgG; (P) PD on CMV IgG; Abbreviations: EBV, Epstein-Barr virus; CMV, cytomegalovirus; HSV, herpes simplex; EBNA1, Epstein-Barr virus nuclear antigen-1; VCA, EBV viral capsid antigen; HSV-1, HSV type 1; HSV-2, HSV type 2; PD, periodontitis; MR Mendelian randomization.


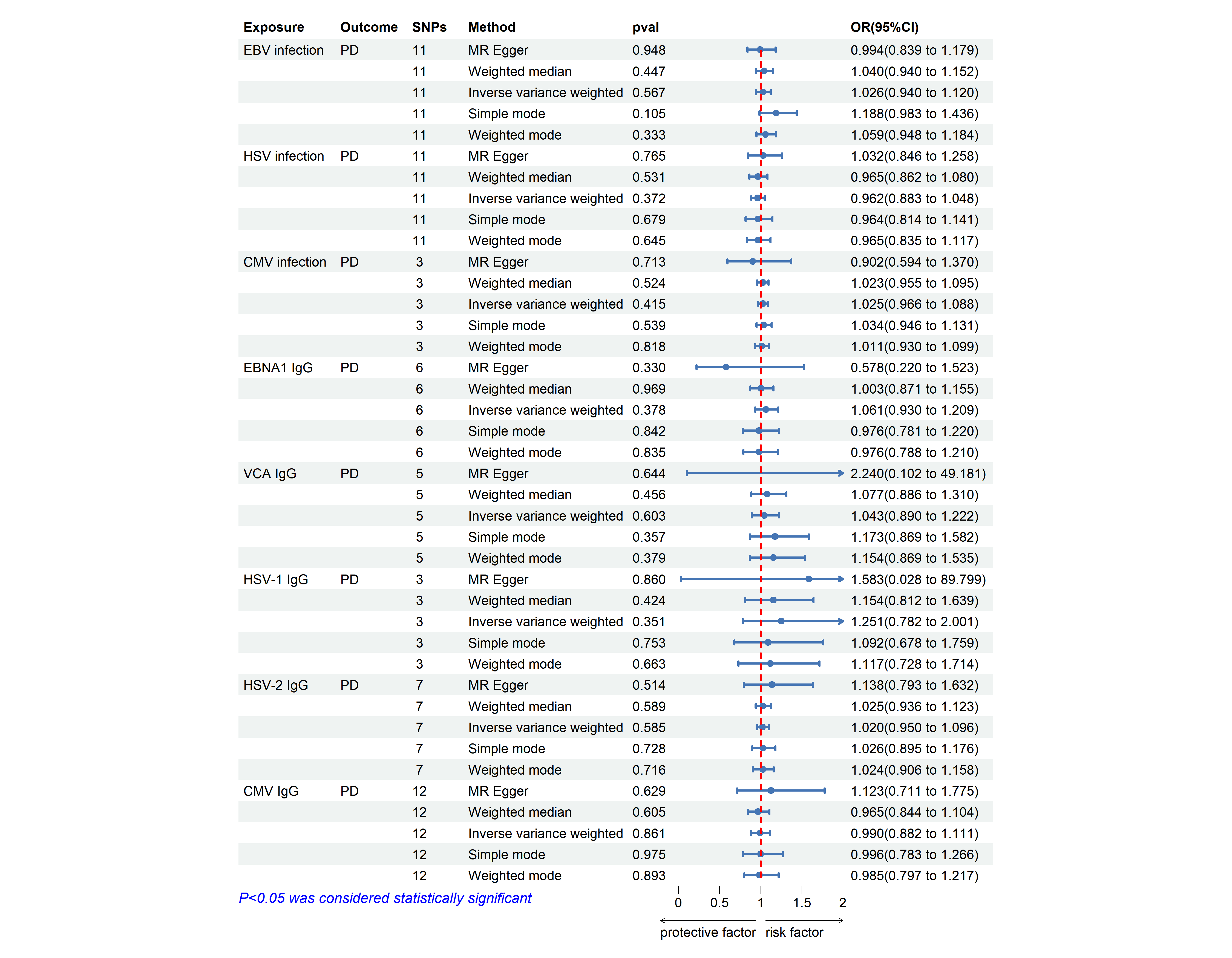


**Fig. S7** Causal estimates between PD and herpesvirus infection given as odds ratios (ORs) and 95% confidence intervals. SNPs, single-nucleotide polymorphisms; *H. pylori*, *Helicobacter pylori*; OR, Odds ratio; 95% CI, 95% confidence intervals; EBV, Epstein-Barr virus; HSV, Herpes simplex virus; CMV, cytomegalovirus; EBNA1, EBV nuclear antigen-1; VCA, EBV virus capsid antigen; HSV-1, HSV type 1; HSV-2, HSV type 2; PD, periodontitis.
